# Low theoretical fidelity hinders the research on health coaching for opioid reduction: A systematic review of randomized controlled trials

**DOI:** 10.1371/journal.pone.0241434

**Published:** 2020-10-29

**Authors:** Natalie A. McNerney, Michael J. Losensky, Madison M. Lash, Kendal R. Rozaieski, Daniela Ortiz, Alessandra N. Garcia, Zachary D. Rethorn

**Affiliations:** Physical Therapy Division, Department of Orthopaedic Surgery, Duke University, Durham, North Carolina, United States of America; University of Maryland School of Medicine, UNITED STATES

## Abstract

**Purpose:**

To systematically review the literature in order to evaluate the effects of health coaching on patients’ reduction of opioid usage and opioid discontinuation. In addition, this systematic review investigated the effects of health coaching on pain intensity, physical function, and quality of life.

**Methods:**

Four electronic databases (PubMed, Embase, Scopus, and PsychINFO) were searched from inception to December 2019. Randomized controlled trials assessing the effects of health coaching interventions in adult patients currently using opioids were included. We considered trials if they included any of the four defined key constructs of health coaching adopted in this review: motivational interviewing, positive psychology, the transtheoretical model, and self-determination theory Independent reviewers screened and selected studies, extracted data, and assessed risk of bias using Revised Cochrane risk-of-bias tool for randomized trials (RoB2) and quality of evidence using Grading, Recommendation, Assessment, Development, and Evaluation (GRADE). The review is registered in the International Prospective Register of Systematic Reviews (PROSPERO) databased as CRD42019136201. It was not possible to perform a meta-analysis due to heterogeneity between included trials.

**Results:**

Eleven studies met our inclusion criteria (n = 4,516 participants). No study assessed all four constructs of health coaching. All eleven studies utilized only one of the constructs, brief motivational interviewing. Thus, we reported our results in terms of motivational interviewing. There is conflicting and very low quality of evidence that brief motivational interviewing may or may not be more effective than education to reduce opioid usage. There is very low quality of evidence that brief motivational interviewing is more effective than educational monthly diaries to reduce opioid use. There is very low to low quality of evidence that brief motivational interviewing is not more effective than no behavioral intervention to reduce opioid use at 6 months follow-up, treatment as usual (TAU) to improve overdose risk behaviors, and TAU to improve physical and psychological health.

**Conclusion:**

There is no direct evidence related to the effect of health coaching on opioid reduction. There is limited, low quality evidence to conclude brief motivational interviewing reduces opioid usage in opioid-dependent patients. Future research should focus on the impact of high theoretical health coaching interventions on opioid usage.

## Introduction

Opioids are well-established analgesics used to treat those with severe acute, cancer related, and surgery related pain [1]. As opioids have been effective in treating these types of pain, physicians now routinely prescribe opioids to treat chronic pain [1]. However, due to the high risk of adverse effects, tolerance build-up, addiction, and overdose, experts have questioned whether opioids should be prescribed to patients with chronic pain [1]. In 2016, the Center for Disease Control and Prevention (CDC) released a call to action aimed to heighten awareness regarding the danger of opioids while addressing the increased need for research focused on creating a guideline for prescribing opioids to those with chronic pain [2]. However, in 2017, opioid use contributed to 47,600 deaths—two-thirds of all overdose-related deaths in the United States [3]. In response to the opioid crisis, as a way to halt the rapid increase in the use of prescription and non-prescription opioids, policy makers have recommended alternative methods to manage pain such as tapering programs, support systems, and non-pharmacologic interventions [3].

Health coaching, a non-pharmacologic intervention, is defined as “a patient-centered process that is based upon the behavior change theory and is delivered by health professionals with diverse backgrounds” [4]. It has been proposed as a strategy to help professionals change health-related behaviors using non-judgmental dialogue that is patient-centered, coupled with realistic goal setting and accountability [5]. Similar to other behavioral interventions, it is important to assess the fidelity of the intervention. Theoretical fidelity is defined as “the degree to which an intervention is implemented as intended by its developers” [6]. According to Moore et al., health coaching consists of four constructs: motivational interviewing, positive psychology, the transtheoretical model, and self-determination theory [7]. Thus, applying the definition of theoretical fidelity to health coaching, high theoretical fidelity health coaching requires the combination and application of all four constructs [4, 7]. Through the implementation of all four constructs, health coaches promote growth, elicit self-motivation, build confidence, and walk through the process of change to help patients and clients become self-determined [7]. Prior research has demonstrated the efficacy of health coaching and the importance of having high theoretical fidelity in behavioral interventions to reduce Hemoglobin A1C levels [8], improve blood pressure, increase physical activity, and improve self-efficacy [9]. A recent meta-analysis concluded that health coaching reduced hospital admissions related to chronic obstructive pulmonary disease and increased patients’ quality of life [10].

In light of the call to action from the CDC, health coaching may be a feasible strategy to combat unnecessary opioid use.[5] Many addicts suffer from a lack motivation, dysfunctional behavior, deficits in self-control, poor social support, and compulsivity [11]. It is clear that opioid addiction is not just physiological but has social and behavioral components as well. Health coaching holds promise in assisting with these behavior changes necessary to reduce opioid use. Despite the effectiveness of medications, such as methadone, research shows that medications must be used in conjunction with appropriate psychosocial treatments to be beneficial [12]. There are no systematic literature summaries which analyze the effect of health coaching on opioid usage when delivered by healthcare professionals. Thus, the purpose of this study is to systematically review the literature to evaluate the effects of health coaching on patients’ reduction of opioid usage and opioid discontinuation. In addition, this systematic review investigated the effects of health coaching on pain intensity, physical function, and quality of life.

## Methods

### Protocol and registration

A protocol for this systematic review was developed prior to the study initiation and is registered in the International Prospective Register of Systematic Reviews (PROSPERO) databased as CRD42019136201 and can be accessed at http://www.crd.york.ac.uk/PROSPERO/display_record.asp?ID=CRD42019136201 (S1 Appendix). This systematic review followed the Preferred Reporting Items for Systematic reviews and Meta-Analyses (PRISMA) and the Cochrane Handbook of Systematic Reviews of Interventions to guide conducting and reporting [13].

### Data sources and search strategy

Systematic literature searches were performed in MEDLINE (PubMed), Embase (Elsevier), Scopus (Elsevier), and PsychINFO (EBSCO) from inception to December 10, 2019. Keywords, Medical Subject Heading (MeSH) terms, and other index terms, as well as combinations and synonyms of these terms, were used to construct the search strategy (S2 Appendix).

### Criteria for considering studies for this review

#### Types of studies

Only randomized controlled trials (RCTs) were included in this review. To be eligible for inclusion, trials consisted of one group receiving at least one element of a health coaching intervention. We included studies published in English, Portuguese, or Spanish. Non-peer reviewed RCTs were not accepted in this review.

#### Types of participants

The population of interest consisted of adults 18 years and older currently using opioids. We did not exclude participants based on health conditions or whether or not they had a substance use disorder diagnosis.

#### Types of interventions

We included trials if the intervention involved health coaching aimed at decreasing opioid usage with a clear focus on changing behavior and attaining health promotion goals. The term health coaching was defined as “a patient-centered process that is based upon behavior change theory and is delivered by health professionals with diverse backgrounds”[4]. Although other definitions of health coaching have been proposed, we chose to use this definition to guide our review process [8, 9, 14]. As prior reviews found heterogenous reporting related to health coaching, we designed a balanced search strategy to capture all potentially relevant articles [9, 15]. We considered trials if they included any of the four key constructs of health coaching: motivational interviewing, positive psychology, the transtheoretical model, and self-determination theory [16]. We included trials that contained any of these elements alone or in combination with other interventions. We did not consider trials to be eligible if they only included written advice without any individually tailored discussion relative to opioid use. We defined an intervention to have high theoretical fidelity if it utilized all four constructs of health coaching. Conversely, if an intervention did not contain all four constructs, we classified it as low theoretical fidelity [4, 7].

#### Comparison and control conditions

Comparison and control conditions accepted in this review included no treatment, minimal intervention (i.e. brief education treatment/booklets), virtual treatment (i.e. phone or online conversations), treatment as usual or standard care (i.e. same medication regimen), or no intervention.

#### Types of outcomes

Our primary outcomes consisted of opioid usage prior to and following a health coaching intervention. We defined opioid use as any current use of opioids, including previous non- fatal opioid overdoses. We included studies if opioids were used independently or if they were used in combination with other drugs. Secondary outcomes included physical function, pain intensity, and quality of life to encompass physiological and psychological health outcomes.

### Study selection

Two trained reviewers (NAM and MJL) independently screened titles and abstracts. Full texts of potentially eligible articles were assessed against predetermined eligibility criteria by the same reviewers. Disagreements were resolved by discussion, or if necessary, arbitration by a third reviewer (DO). Agreement between reviewers on the inclusion of titles/abstracts and full-text articles were quantified using Cohen’s kappa [17].

### Data extraction

Data from the included studies were extracted independently using a predetermined data extraction form by two reviewers (NAM and DO). Disagreements in extraction were resolved by discussion or if necessary, a third reviewer (MML). Data extracted included: population characteristics (e.g., country, setting, number of participants, gender, age, health condition), intervention used, comparison intervention, number of sessions and duration of treatment, and primary and secondary outcomes with their respective time frame follow-up. When necessary, we contacted primary authors via email for missing information and followed up twice at two weeks and at four weeks after the initial email.

### Assessment of risk of bias

Two trained independent reviewers (MML and KRR) assessed the risk of bias for each included study using the Revised Cochrane risk-of-bias tool for randomized trials (RoB2) [18]. The tool is considered to be a valid and reliable measurement of methodological quality in RCTs [18]. RoB2 analyzes sequence generation; allocation concealment; blinding of participants, personnel, and outcome assessment; incomplete outcome data; selective reporting of outcomes, and other sources of bias. RoB2 incorporates an algorithm to calculate the risk of bias based on questions and responses to the above domains. Each domain was scored in one of three categories: “high risk of bias,” “low risk of bias,” or “unclear.” We classified the risk of bias into these categories according to the results generated from the RoB2 algorithm. We classified studies as having a “high risk of bias” if the authors judged any domain to be high risk. We classified studies as having “low risk of bias” if the authors judged all domains to be low risk, and we classified studies as “some concern” if at least one domain raises some concern but no domains are high risk [18]. Disagreements were resolved by discussion or if necessary, a third reviewer (NAM).

### Data synthesis

The effects of the intervention were summarized for the following time periods: short-term follow up (outcomes measured closest to four weeks after randomization), intermediate follow up (outcomes measured closest to six months after randomization) and long-term follow-up (outcomes measured closest to 12 months after randomization). The authors qualitatively synthesized the data using means and standard deviations for continuous variables and odds ratios and 95% confidence intervals for dichotomous variables. We were unable to perform a meta-analysis due to heterogeneity between included trials. We conducted a Grading, Recommendation, Assessment, Development, and Evaluation (GRADE) analysis to assess the quality of the evidence [19]. As we could not perform a meta-analysis, we did not consider two categories in our GRADE assessment: inconsistency of results (which refers to unexplained heterogeneity of results when synthesized together) [20] and publication bias (which is recommended when there are at least ten studies in comparison) across all studies that measure a particular outcome [21]. Thus, we used a modified version of GRADE with the inclusion of three of the five categories: 1) limitations in the study design (risk of bias) [bias analyzes limitations in the design and implementation of the study] [22], 2) indirectness of the evidence [refers to the inability to generalize the population, intervention, comparison, and outcome in the study] [23], and 3) imprecision [accounts for insufficient data by examining the number of participants and the width of confidence intervals for each outcome] [24]. The certainty of evidence in each study was downgraded one level according to the performance of the studies against these three factors. After a consensus meeting amongst the research authors, we decided upon the following criteria outlined in S3 Appendix [25].

## Results

### Study selection and general characteristics of included studies

**Fig 1:**
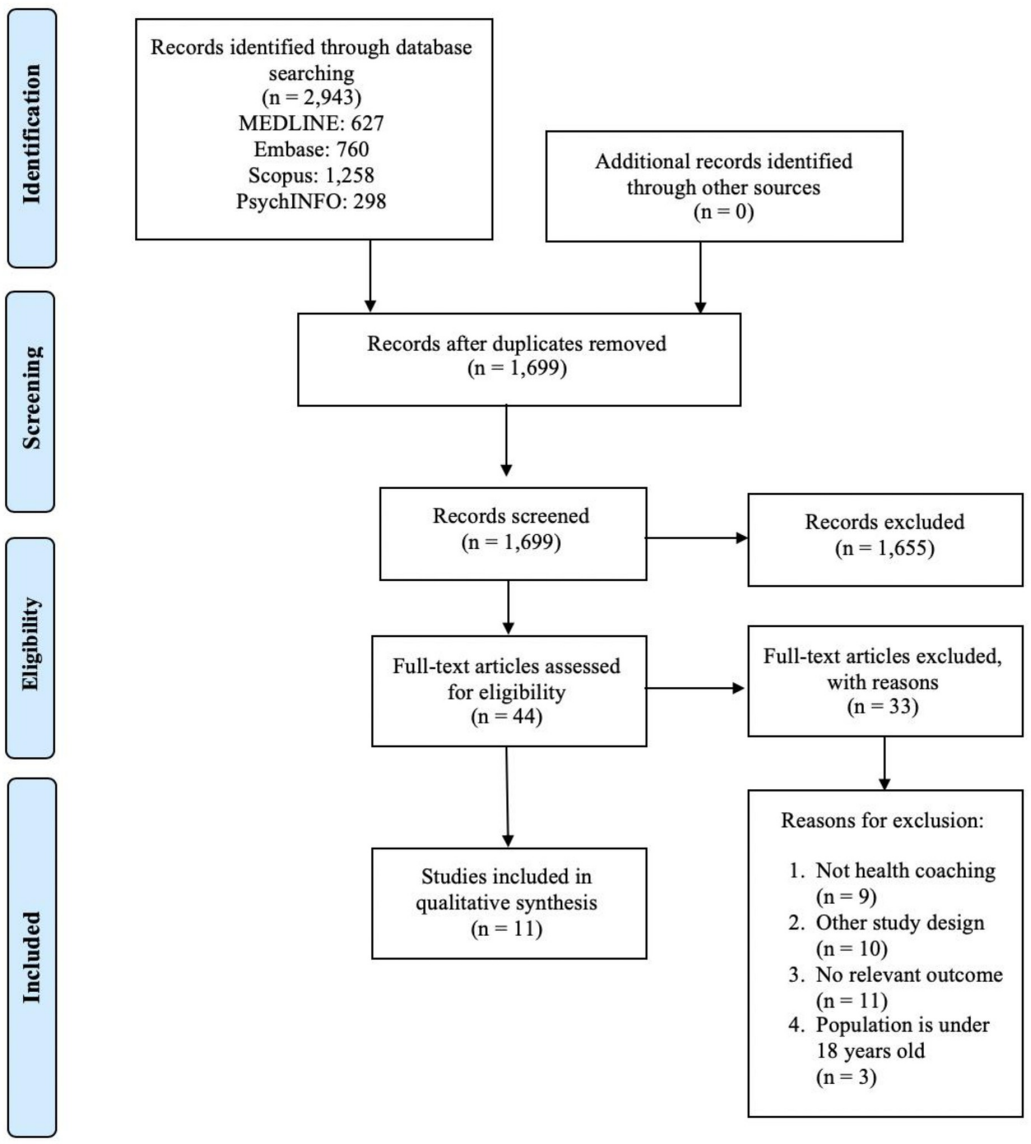
Flow of study selection and review process.

A total of 1,699 studies were identified after removing duplicates (Fig 1). After screening 44 full-text articles, eleven trials met the inclusion criteria and were included in this review [26–36]. Reasons for study exclusion included: other study design (n = 10), interventions failed to meet the definition of health coaching (n = 9), the patient population was under 18 years of age (n = 3), or no relevant outcome was measured (n = 11). A complete list of reasons for exclusion is available in S4 Appendix. The reviewers had a high percentage of agreement during the title and abstract screening (Cohen’s kappa = 86.25%) and during full-text screening (Cohen’s kappa = 80.48%). All included studies were published between 1995 and 2017. The eleven trials included in this review were conducted in five countries. Seven studies were conducted in the United States [27–30, 32, 33, 36] while single trials were conducted in Norway [34], Scotland [31], Australia [26], and China [35] (S5 Appendix).

### Participants

A total of 4,516 participants were included in our review (average sample size = 411 participants). Participants in the studies were 64.24% male with an average age of 38.23 years. Two studies included patients in receiving methadone treatment [26, 28], one study examined opioid usage amongst participants with musculoskeletal disorders [30], two studies included participants with non-acute health problems [27, 36], one study included participants with a prior non-fatal overdose [32], one study included patients seeking psychiatric care [34], one study included participants who receive naloxone from a naloxone distribution center [32], and one study included participants who received prescribed methadone from pharmacists [31]. Two studies included participants from the emergency department [29, 34], four studies included participants from outpatient clinics [27, 30, 33, 36], and three studies included participants in addiction treatment center [26, 28, 35] (S5 Appendix).

### Intervention and comparisons

Though we found studies that utilized individual constructs of health coaching, there was no direct evidence related to the effect of health coaching on opioid reduction. No study assessed all four constructs of health coaching, and each study had low theoretical fidelity as defined above. All eleven studies utilized only one of the constructs, motivational interviewing. Therefore, we reported our results in terms of motivational interviewing. Ten studies analyzed the effects that brief motivational interviewing had on the reduction of opioid misuse and opioid risk behaviors [26–31, 33–36], and one study analyzed the impact of brief motivational interviewing in reducing opioid overdose [32]. Health coaching interventions were administered in the trials investigating the effectiveness of brief motivational interviewing on opioid use reduction in opioid-dependent patients. Forms of motivational interviewing included repeated-dose brief behavioral interventions addressing opioid overdose and related risk behaviors (REBOOT) [32], brief interventions (BI) [29, 36], an adaptation of motivational interviewing (MOTIV) [27], and comprehensive psychosocial intervention (CPI), which included Cognitive Behavioral Therapy (CBT) and brief motivational interviewing techniques [35].

The interventions were administered by trained therapists [28, 34], trained clinicians [33], counselors [27, 32], trained pharmacists [31], psychologists [30], research assistants [26, 29], peers [36], and social workers [35]. Comparisons administered in the studies included treatment as usual [29, 31–35], monthly diaries [30], educational information [26, 28, 36], or no intervention [27]. Treatment, as usual, consisted of general psychotherapy and pharmacotherapy [34], standard intakes and evaluation [33], information about substance use disorder treatment [32], prescribed methadone [31], current opioid regimens [30], and monthly visits by a social worker [35]. Educational information included an educational handout discussing treatment options and harm reduction information [36], a nurse-led hepatitis health promotion program [28], and an opiate drug informational booklet [26]. Duration of the intervention and number of sessions ranged from 30 minutes [29] to 60 minutes [35] and one session [26, 27, 29, 31, 33, 36] to multiple sessions over 16 months [32]. Four studies conducted short term follow-up [29, 32–34], four studies carried out intermediate follow-up [27, 30, 31, 36], one study utilized long term follow-up [35], and two studies did not provide this information [26, 28] (S5 Appendix).

### Primary and secondary outcomes

Eleven studies analyzed our primary outcome and reported opioid usage of participants through self-reported questionnaires or outcomes [26–36]. Three studies utilized urine screens [30, 33, 35], two studies took hair samples [27, 36], and two studies reported through structured interviews [26, 31]. Eight studies reported opioid use in number of days [26–29, 31, 33, 34, 36], three studies reported opioid usage in months [28, 30, 35], and one study reported number of opioid overdoses [32]. Two studies analyzed our secondary outcomes and looked at quality of life (physical health, psychological health, and pain intensity amongst participants) [31, 35] (S5 Appendix).

### Risk of bias

**Fig 2:**
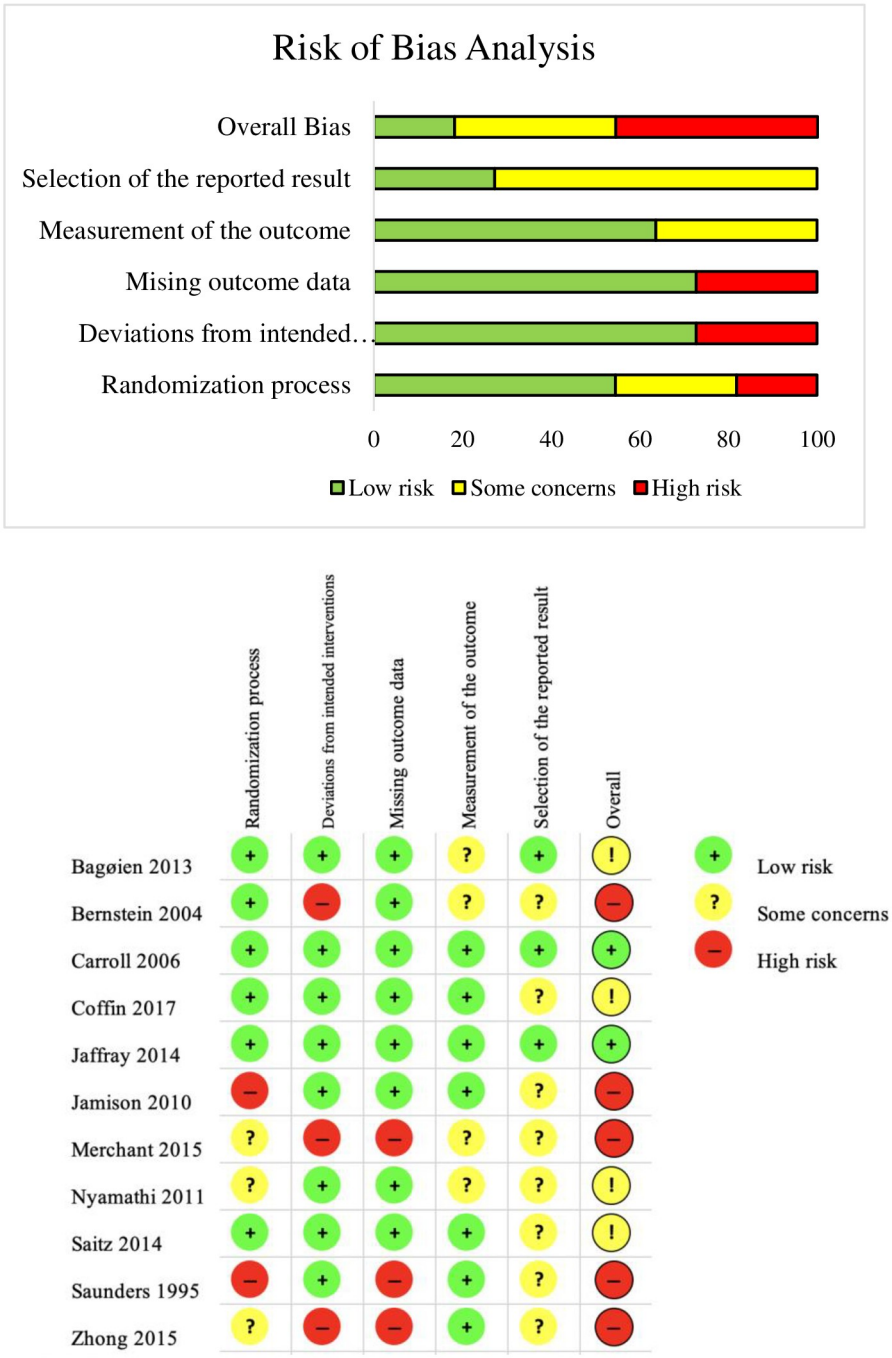
Composite risk of bias.

The results of the risk of bias are shown in Fig 2. Five studies (45.5%) had a high risk of bias [26, 29, 30, 35, 36], two studies (18.2%) had a low risk of bias [31, 33], and four studies (36.4%) showed some concern [27, 28, 32, 34]. Major limitations to study quality included the selection of the reported result, deviations from the intended intervention, and missing outcome data. Eight studies showed some concern in the selection of the reported result [26–30, 32, 35, 36], while three studies had high risk in deviations from the intended intervention [29, 35, 36] and missing outcome data [26, 29, 35].

### The effects of brief motivational interviewing on opioid usage

The effects of brief motivational interviewing delivered were measured in different ways. In this review, the authors divided the outcomes of the included studies into four domains: opioid use, overdose risk behaviors, physiological health, and psychological health (Table 1).

**Table 1:** Effects of brief motivational interviewing interventions and quality of evidence.

Study	Country	Population	N	Intervention	Comparator	Outcome	Time frame	Effect estimate—mean [SD]	Effect estimate—OR [95% CI]	Quality of evidence
**Primary Outcomes**										
Bagøien et al., 2013	Norway	Psychiatric Inpatient	130	MI + TAU	TAU	Opioid Use	Short	0.60 [-1.40, 2.60]	------	⊕⊝⊝⊝ Very low^1^^,^^2^^,^^3^
Bernstein et al., 2005	USA	Primary Care	349	MI	Education	Opioid Use	Intermediate	------	1.64 [1.05, 2.56]	⊕⊝⊝⊝ Very low^1^^,^^3^
Carroll et al., 2006	USA	Community Addiction Treatment Centers	336	MI evaluation	Standard evaluation	Opioid Use	Short	0.20 [-2.47, 2.87]	------	⊕⊕⊝⊝ Low^2^^,^^3^
Jaffray et al., 2014	Scotland	Community pharmacies	335	MI training for pharmacists	Usual pharmacist care	Opioid Use	Intermediate	------	1.05 [0.66, 1.66]	⊕⊕⊝⊝ Low^2^^,^^3^
Jamison et al., 2010	USA	Non-cancer back pain	58	MI package	Monthly diary	Opioid Use	Intermediate	------	8.54 [1.53, 47.71]	⊕⊝⊝⊝ Very low^1^^,^^3^
Merchant et al., 2015	USA	Emergency Room	633	MI	TAU	Opioid Use	Short	------	0.90 [0.64, 1.27]	⊕⊝⊝⊝ Very low^1^^,^^2^^,^^3^
Saitz et al., 2014	USA	Primary care	278	MI	No treatment control	Opioid Use	Intermediate	------	1.28 [0.65, 2.52]	⊕⊝⊝⊝ Very low^1^^,^^2^^,^^3^
			269	MI	Brief interview	Opioid Use	Intermediate	------	0.70 [0.37, 1.30]	⊕⊝⊝⊝ Very low^1^^,^^2^^,^^3^
Zhong et al., 2015	China	Community rehabilitation program	173	MI + CBT	TAU	Opioid Use	Long	------	1.08 [0.54, 2.17]	⊕⊝⊝⊝ Very low^1^^,^^2^^,^^3^
**Secondary Outcomes**										
Jaffray et al., 2014	Scotland	Community pharmacies	335	MI training for pharmacists	Usual pharmacist care	Physical health	Intermediate	1.00 [-0.60, 2.60]	------	⊕⊕⊝⊝ Low^2^^,^^3^
						Psychological health	Intermediate	0.60 [-1.64, 2.84]	------	⊕⊕⊝⊝ Low^2^^,^^3^
Zhong et al., 2015	China	Community rehabilitation program	156	MI + CBT	TAU	Physical functioning	Long	6.78 [0.87, 12.69]	------	⊕⊝⊝⊝ Very low^1^^,^^2^^,^^3^
						Pain	Long	9.13 [-15.39, 33.65]	------	⊕⊝⊝⊝ Very low^1^^,^^2^^,^^3^

• Three studies (Coffin et al., Nyamathi et al., Saunders et al.) were not included in this table due to insufficient quantitative data and are all very low quality of evidence.^1,2,3^

GRADE (Grading of Recommendations Assessment, Development, and Evaluation) Quality of Evidence and Definitions:[37]

• *High quality (*⊕⊕⊕⊕): Further research is very unlikely to change our confidence in the estimate of effect

• *Moderate quality (*⊕⊕⊕⊝): Further research is likely to have an importance impact on our confidence in the estimate of effect and may change the estimate

• *Low quality (*⊕⊕⊝⊝): Further research is likely to have an important impact on our confidence in the estimate of effect and is likely to change the estimate

• *Very low quality (*⊕⊝⊝⊝): Any estimate of effect is very uncertain and we have little confidence in the estimate of effect

^1^Downgraded one level due to limitations in risk of bias

^2^Downgraded one level due to imprecision

^3^Downgraded one level due to indirectness

#### Opioid use

There is very [29, 32, 34, 35] to low quality of evidence [31, 33] that brief motivational interviewing is not more effective to reduce opioid use compared to treatment as usual (Table 1). Each study concluded there were no significant differences in substance use between the experimental and control groups as early as 30 days [31] and as long as 16 months follow-up [32] (Table 1).

There is conflicting evidence on the effect of brief motivational interviewing compared to education on opioid use reduction. There is very low quality of evidence that brief motivational interviewing may be more effective than education (an educational handout discussing treatment options and harm reduction information) to reduce opioid use [36]. Berstein et al. reported that at 6 months, the group that received the brief motivational interviewing intervention had a greater reduction in opioid use than the group that received educational information (OR = 1.64, 95% CI = 1.05, 2.56) [36]. There is also very low quality of evidence that brief motivational interviewing may not be more effective than education (a nurse-led hepatitis health promotion program or an opiate drug informational booklet) to reduce opioid use [26, 28]. Nyamathi reported that both brief individual motivational interviewing and group motivational interviewing were effective in decreasing average daily drug intake but did not provide data to support this statement [28]. Saunders reported that brief motivational interviewing reduced client self-efficacy but did not significantly decrease opioid dependence [26]. Attempts to contact the primary author for additional data were unsuccessful.

There is very low quality of evidence that brief motivational interviewing may be more effective than monthly diaries to reduce opioid [30]. This study concluded that there was a significant reduction in substance use of the intervention group compared to the control group at 6 months (OR = 8.54, 95% CI = 1.53,47.71) [30].

There is very low quality of evidence that brief motivational interviewing may not be more effective than no behavioral intervention to reduce opioid use at 6 months follow-up [27].

#### Overdose risk behaviors

There is very low quality of evidence that brief motivational interviewing may not be more effective than treatment as usual to improve overdose risk behaviors [32]. Coffin reported that the mean number of overdose events decreased significantly among REBOOT participants compared to TAU [32], but the study did not provide sufficient data to draw conclusions on the effect of the intervention. Attempts to contact the author for additional data were unsuccessful.

#### Physiological and physical health

There is low quality of evidence that brief motivational interviewing may not be more effective than TAU at improving physical health and psychological health [31]. There is very low quality of evidence that a combination of brief motivational interviewing and cognitive behavioral therapy may be more effective than TAU at improving physical function (mean = 6.78, SD = 0.87, 12.69) [35]. This same study reported that brief motivational interviewing may not be more effective than TAU at improving pain intensity amongst participants.

## Discussion

The principal finding of this review is that brief motivational interviewing is not superior to other interventions to reduce opioid usage in opioid-dependent patients. The aim of this systematic review was to examine the effect of health coaching on opioid usage. However, no study included in this review utilized the four constructs of the predefined definition of health coaching. Because we conducted a balanced search strategy, we found eleven studies that examined one theoretical construct, motivational interviewing. We classified an intervention as having high theoretical fidelity if it included all four constructs of health coaching. All included studies had low theoretical fidelity. Of the eleven studies included in this review, only three studies reported statistically significant results [30, 35, 36]. Each of these studies had high risk of bias and very low quality of evidence. One study had a small effect size [36], and two studies had a moderate effect size [30, 35]. Although these findings were statistically significant, our confidence in these results is limited.

Most of the studies in this review utilized extremely brief motivational interviewing sessions. The studies provided single sessions or sessions that lasted less than 60 minutes. Research shows that variability exists in accepted intervention lengths that range from brief motivational interviewing sessions of 5 to 15 minutes to a mean intervention time of 207 minutes [38, 39]. Although variability exists in the literature, we recognize that only including brief motivational interviewing interventions is a major limitation to our review. These brief motivational interviewing interventions should not be misinterpreted to suggest that motivational interviewing as a whole in not effective in reducing opioid usage.

While brief motivational interviewing was not found to be superior to other interventions for reduction of opioid usage, previous studies have investigated the effectiveness of other behavioral interventions, such as cognitive behavioral therapy (CBT), on the reduction of opioid usage. CBT has been shown to increase abstinence from drugs in those addicted to prescription opioids [40] and to be superior to methadone drug counseling at increasing abstinence from non-prescription opioids [41]. These results, while promising, are in contrast to our results. Studies including CBT consistently demonstrated high theoretical fidelity while all the studies included in this review only included one of the four aspects of health coaching and therefore have significantly lower fidelity [42]. This difference between the two types of behavioral interventions could possibly be the reason for the lack of significant, high quality findings, and is an avenue for future research in health coaching.

This systematic review has several strengths. It is the first to summarize the effects of the health coaching intervention on opioid-dependent patients. We utilized a comprehensive search strategy developed by a medical librarian and a pre-specified data extraction form. We followed PRISMA guidelines and pre-registered the review with PROSPERO. No changes were made to the initial protocol. Two individuals performed data extraction, and two other members of the team checked their work to ensure all necessary data was found. The systematic review used the Cochrane RoB2, a robust tool of quality assessment which was completed independently by two individuals to avoid bias. It also utilized GRADE to further synthesis the quality of evidence from the included studies. Although this review presents low quality evidence, it was well designed and methodologically strong.

This review is not without limitations. We included randomized controlled trials and studies in English, Spanish, and Portuguese. As a result, potential papers in other languages that included aspects of health coaching may have been excluded. We only included randomized controlled trials in this review which may have introduced publication bias. However, we believe this to be unlikely as the majority of included trials did not find statistically significant improvements following brief motivational interviewing interventions. The quality of evidence for the included articles was concerning due to limitations in risk of bias, imprecision, and indirectness. This has implications as it lowers our confidence in the results. Three studies combined methadone therapy and brief motivational interviewing as the treatment to its participants [26, 28, 31]. We recognize that this is a limitation to our review, since these two interventions used in combination may contribute to greater positive outcomes than one treatment used individually. Both methadone therapy and motivational interviewing are extremely complicated interventions, and nearly impossible to tease apart when used in conjunction with each other. Thus, we were unable to separate one intervention from the other. Due to study heterogeneity, we had eleven single point estimates, so we were unable to perform a meta-analysis. This prevented us from synthesizing the results to make further comparisons.

Our systematic review highlights the dearth of published literature related to health coaching and its effect on opioid use. Future research should utilize theoretically robust health coaching interventions using all four of the theoretical constructs, as the literature presently reviewed has only focused solely on brief motivational interviewing. Fidelity measures have been developed for motivational interviewing but have not been utilized in health coaching [43]. Subsequent studies should focus on how fidelity measures could be formulated and applied to health coaching interventions to help more clearly define high theoretical health coaching. As apparent in the included studies, behavioral interventions are difficult to control. It is challenging to administer an appropriate intervention to meet the definition of health coaching while having high theoretical fidelity. Future research should account for this complication and focus on how behavioral interventions could help patients with chronic pain and musculoskeletal problems reduce opioid usage.

## Conclusion

There was no direct evidence on the effect of health coaching on opioid reduction. Our systematic review suggests that there is limited, very low quality of evidence that brief motivational interviewing is not superior to other interventions or treatment as usual to reduce opioid usage. Due to the low quality of evidence and lack of theoretical fidelity, we must interpret these results with caution. Future research should focus on the administration and the impact of high theoretical health coaching interventions on opioid usage.

## Supporting information

S1 Checklist (DOC)

S1 Appendix International Prospective Register of Systematic Reviews (PROSPERO). (DOCX)

S2 Appendix Search terms used for electronic databases. (DOCX)

S3 Appendix The GRADE approach to evidence synthesis. (DOCX)

S4 Appendix List of excluded studies/reasons for exclusion. (DOCX)

S5 Appendix A comprehensive description of each study. (DOCX)
