# Supplementary material for: Low theoretical fidelity hinders the research on health coaching for opioid reduction: A systematic review of randomized controlled trials
Source: PLoS One. 2020 Oct 29;15(10):e0241434. doi: 10.1371/journal.pone.0241434 (PMC7595321; doi:10.1371/journal.pone.0241434)
Supplement: S1 Appendix — (DOCX) [file pone.0241434.s002.docx]

**S1 Appendix. International Prospective Register of Systematic Reviews (PROSPERO)**


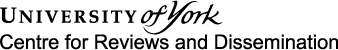


Systematic review

# * Review title.

Give the working title of the review, for example the one used for obtaining funding. Ideally the title should state succinctly the interventions or exposures being reviewed and the associated health or social problems. Where appropriate, the title should use the PI(E)COS structure to contain information on the Participants, Intervention (or Exposure) and Comparison groups, the Outcomes to be measured and Study designs to be included.

The effects of health coaching intervention to reduce opioid use in patients with general health disorders

# Original language title.

For reviews in languages other than English, this field should be used to enter the title in the language of the review. This will be displayed together with the English language title.

# * Anticipated or actual start date.

Give the date when the systematic review commenced, or is expected to commence. 28/05/2019

# * Anticipated completion date.

Give the date by which the review is expected to be completed. 06/12/2019

# * Stage of review at time of this submission.

Indicate the stage of progress of the review by ticking the relevant Started and Completed boxes. Additional information may be added in the free text box provided.

Please note: Reviews that have progressed beyond the point of completing data extraction at the time of initial registration are not eligible for inclusion in PROSPERO. Should evidence of incorrect status and/or completion date being supplied at the time of submission come to light, the content of the PROSPERO record will be removed leaving only the title and named contact details and a statement that inaccuracies in the stage of the review date had been identified.

This field should be updated when any amendments are made to a published record and on completion and publication of the review. If this field was pre-populated from the initial screening questions then you are not able to edit it until the record is published.

The review has not yet started: No

**Review stage Started Completed**

Preliminary searches Yes No

Piloting of the study selection process No No

Formal screening of search results against eligibility criteria No No

Data extraction No No

Risk of bias (quality) assessment No No

Data analysis No No

Provide any other relevant information about the stage of the review here (e.g. Funded proposal, protocol not yet finalised).

# * Named contact.

The named contact acts as the guarantor for the accuracy of the information presented in the register record. Natalie McNerney

Email salutation (e.g. "Dr Smith" or "Joanne") for correspondence:

Miss McNerney

# * Named contact email.

Give the electronic mail address of the named contact. [natalie.mcnerney@duke.edu](mailto:natalie.mcnerney@duke.edu)

# Named contact address

Give the full postal address for the named contact. 200 Glengary Court, Durham, NC 27707

# Named contact phone number.

Give the telephone number for the named contact, including international dialling code. 954-612-5534

# * Organisational affiliation of the review.

Full title of the organisational affiliations for this review and website address if available. This field may be completed as 'None' if the review is not affiliated to any organisation.

Duke University School of Medicine Division of Physical Therapy

Organisation web address:

1. * Review team members and their organisational affiliations.

Give the title, first name, last name and the organisational affiliations of each member of the review team.

Affiliation refers to groups or organisations to which review team members belong.

Miss Natalie McNerney. Duke University School of Medicine Division of Physical Therapy, Department of Orthopaedic Surgery

Mr Michael Losensky. Duke University School of Medicine Division of Physical Therapy, Department of Orthopaedic Surgery

Miss Daniela Ortiz. Duke University School of Medicine Division of Physical Therapy, Department of Orthopaedic Surgery

Miss Kendal Rozaieski. Duke University School of Medicine Division of Physical Therapy, Department of Orthopaedic Surgery

Miss Madison Lash. Duke University School of Medicine Division of Physical Therapy, Department of Orthopaedic Surgery

Dr Zachary Rethorn. Duke University School of Medicine Division of Physical Therapy, Department of Orthopaedic Surgery

Dr Alessandra Narciso Garcia Trepte. Duke University School of Medicine Division of Physical Therapy, Department of Orthopaedic Surgery

# * Funding sources/sponsors.

Give details of the individuals, organizations, groups or other legal entities who take responsibility for initiating, managing, sponsoring and/or financing the review. Include any unique identification numbers assigned to the review by the individuals or bodies listed.

None

# * Conflicts of interest.

List any conditions that could lead to actual or perceived undue influence on judgements concerning the main topic investigated in the review.

None

# Collaborators.

Give the name and affiliation of any individuals or organisations who are working on the review but who are not listed as review team members.

Ms Karen Barton. Duke University School of Medicine Library

# * Review question.

State the question(s) to be addressed by the review, clearly and precisely. Review questions may be specific or broad. It may be appropriate to break very broad questions down into a series of related more specific questions. Questions may be framed or refined using PI(E)COS where relevant.

This project will identify the effects of health coaching treatments on opioid use in adults.

# * Searches.

State the sources that will be searched. Give the search dates, and any restrictions (e.g. language or publication period). Do NOT enter the full search strategy (it may be provided as a link or attachment.)

We will search the following electronic bibliographic databases: PubMed, Embase, Scopus, and PsycINFO from inception up to June 5, 2019, without language restrictions. We will combine search terms related to health coaching and opioids. The search terms will be adjusted for each of the databases previously mentioned. The searches will be re-run just before the final analyses and further studied retrieved for inclusion. In addition to the electronic database search, other potentially eligible reviews will be identified by

reviewing the references lists of each eligible review.

# URL to search strategy.

Give a link to a published pdf/word document detailing either the search strategy or an example of a search strategy for a specific database if available (including the keywords that will be used in the search strategies), or upload your search strategy.Do NOT provide links to your search results.

[https://www.crd.york.ac.uk/PROSPEROFILES/136201_STRATEGY_20190609.pdf](http://www.crd.york.ac.uk/PROSPEROFILES/136201_STRATEGY_20190609.pdf)

Alternatively, upload your search strategy to CRD in pdf format. Please note that by doing so you are consenting to the file being made publicly accessible.

Do not make this file publicly available until the review is complete

# * Condition or domain being studied.

Give a short description of the disease, condition or healthcare domain being studied. This could include health and wellbeing outcomes.

Pain conditions: musculoskeletal pain, general pain and/or general health disorders

# * Participants/population.

Give summary criteria for the participants or populations being studied by the review. The preferred format includes details of both inclusion and exclusion criteria.

Adult participants (older than or equal to 18 years old), either sex with pain, with or without a substance use disorder diagnosis.

# * Intervention(s), exposure(s).

Give full and clear descriptions or definitions of the nature of the interventions or the exposures to be reviewed.

Health coaching was defined as “a client-centered process to facilitate and empower the client to achieve self-determined goals related to health and wellness.” Trials could include a variety of delivery methods including face-to-face coaching, telephone-based coaching, or coaching using electronic communication such as text message or e-mail. Studies will be excluded if they do not include opioid reduction as one of the outcomes of interest.

# * Comparator(s)/control.

Where relevant, give details of the alternatives against which the main subject/topic of the review will be compared (e.g. another intervention or a non-exposed control group). The preferred format includes details of both inclusion and exclusion criteria.

Expected comparisons include advice or education, exercise therapy, manual therapy, psychological interventions such as ACT, usual care, or a wait-list control.

# * Types of study to be included.

Give details of the types of study (study designs) eligible for inclusion in the review. If there are no restrictions on the types of study design eligible for inclusion, or certain study types are excluded, this should be stated. The preferred format includes details of both inclusion and exclusion criteria.

Eligible study designs include randomized controlled trials. If an eligible study will be published in a language other than those that the authors could read (English, Portuguese, and Spanish), all possible efforts will be made to get a translation. If that will not feasible, the articles will be excluded. We will exclude any study with non-human participants.

# Context.

Give summary details of the setting and other relevant characteristics which help define the inclusion or exclusion criteria.

# * Main outcome(s).

Give the pre-specified main (most important) outcomes of the review, including details of how the outcome is defined and measured and when these measurement are made, if these are part of the review inclusion criteria.

Primary outcome: opioid use.

Timing and effect measures

Effects will be summarized for the following time periods: short-term follow-up (outcomes measured closest to four weeks after randomization), intermediate follow-up (outcomes measured closest to six months after randomization) and long-term follow-up (outcomes measured closest to 12 months after randomization).

# * Additional outcome(s).

List the pre-specified additional outcomes of the review, with a similar level of detail to that required for main outcomes. Where there are no additional outcomes please state ‘None’ or ‘Not applicable’ as appropriate

to the review

Secondary outcomes: opioid withdrawal symptoms, pain intensity, physical function, and quality of life.

Timing and effect measures

Effects will be summarized for the following time periods: short-term follow-up (outcomes measured closest to four weeks after randomization), intermediate follow-up (outcomes measured closest to six months after randomization) and long-term follow-up (outcomes measured closest to 12 months after randomization).

# * Data extraction (selection and coding).

Describe how studies will be selected for inclusion. State what data will be extracted or obtained. State how this will be done and recorded.

Two review authors will independently screen all titles and/or abstract of studies retrieved using the search strategy. The full text of these potentially eligible studies will be independently assessed for eligibility by two review authors. Disagreements will be resolved through discussion with a third reviewer. If an eligible study will be published in a language other than those which the authors could read (English, Portuguese and Spanish) all possible efforts will be made to get a translation and when it was not feasible the articles will be excluded.

Extracted information will include: publication data (authors, journal and year); study setting; study

population; number and demographic characteristics of participants; details of the intervention and control conditions; risk of bias; presence of co-interventions; risk of bias; measures used to assess primary and secondary outcomes; time of assessment and number of participants assessed; results for the assessment of primary and secondary outcomes. Two review authors will extract data independently, discrepancies will be identified and resolved through discussion (with a third author where necessary). Missing data will be requested from study authors.

# * Risk of bias (quality) assessment.

Describe the method of assessing risk of bias or quality assessment. State which characteristics of the studies will be assessed and any formal risk of bias tools that will be used.

Two authors will independently assess the risk of bias in the included studies, a third author will then reassess the risk of bias in the studies. The authors will assess the methodological and reporting quality to give the reader a broader perspective. The authors will utilize the Cochrane Risk of Bias to assess the seven domains including; random sequence, allocation concealment, blinding of participants and researchers, blinding of outcome assessment, incomplete outcome data handling, selective reporting issues, and other.

# * Strategy for data synthesis.

Provide details of the planned synthesis including a rationale for the methods selected. This **must not be generic text** but should be **specific to your review** and describe how the proposed analysis will be applied to your data.

We will provide a narrative synthesis of the findings from the included studies, structured around the study setting, population condition, characteristics of participants, outcomes and intervention content.

We plan to perform a meta-analysis if the data are satisfactorily homogeneous regarding the population, outcome, intervention, comparison, and measures of effects.

# * Analysis of subgroups or subsets.

State any planned investigation of ‘subgroups’. Be clear and specific about which type of study or participant will be included in each group or covariate investigated. State the planned analytic approach.

While subgroup analyses may be undertaken, it is not planned to specify the groups in advance.

# * Type and method of review.

Select the type of review and the review method from the lists below. Select the health area(s) of interest for your review.

Type of review Cost effectiveness No

Diagnostic No

Epidemiologic No

Individual patient data (IPD) meta-analysis No

Intervention No

Meta-analysis No

Methodology No

Narrative synthesis Yes

Network meta-analysis No

Pre-clinical No

Prevention No

Prognostic No

Prospective meta-analysis (PMA) No

Review of reviews No

Service delivery No

Synthesis of qualitative studies No

Systematic review Yes

Other No

Health area of the review Alcohol/substance misuse/abuse Yes

Blood and immune system No

Cancer Yes

Cardiovascular Yes

Care of the elderly No

Child health No

Complementary therapies No

Crime and justice No

Dental No

Digestive system No

Ear, nose and throat Yes

Education Yes

Endocrine and metabolic disorders Yes

Eye disorders No

General interest No

Genetics No

Health inequalities/health equity Yes

Infections and infestations No

International development No

Mental health and behavioural conditions Yes

Musculoskeletal Yes

Neurological Yes

Nursing No

Obstetrics and gynaecology No

Oral health No

Palliative care Yes

Perioperative care No

Physiotherapy Yes

Pregnancy and childbirth No

Public health (including social determinants of health) Yes

Rehabilitation Yes

Respiratory disorders Yes

Service delivery No

Skin disorders No

Social care Yes

Surgery

Yes

Tropical Medicine No

Urological No

Wounds, injuries and accidents Yes

Violence and abuse Yes

# Language.

Select each language individually to add it to the list below, use the bin icon to remove any added in error. English

Portuguese-Brazil Spanish

There is not an English language summary

# * Country.

Select the country in which the review is being carried out from the drop down list. For multi-national collaborations select all the countries involved.

United States of America

# Other registration details.

Give the name of any organisation where the systematic review title or protocol is registered (such as with The Campbell Collaboration, or The Joanna Briggs Institute) together with any unique identification number assigned. (N.B. Registration details for Cochrane protocols will be automatically entered). If extracted data will be stored and made available through a repository such as the Systematic Review Data Repository (SRDR), details and a link should be included here. If none, leave blank.

# Reference and/or URL for published protocol.

Give the citation and link for the published protocol, if there is one Give the link to the published protocol.

Alternatively, upload your published protocol to CRD in pdf format. Please note that by doing so you are consenting to the file being made publicly accessible.

Yes I give permission for this file to be made publicly available

Please note that the information required in the PROSPERO registration form must be completed in full even if access to a protocol is given.

# Dissemination plans.

Give brief details of plans for communicating essential messages from the review to the appropriate audiences.

We plan to present the results at conferences, in peer-review journals, and on social media.

Do you intend to publish the review on completion?

Yes

# Keywords.

Give words or phrases that best describe the review. Separate keywords with a semicolon or new line. Keywords will help users find the review in the Register (the words do not appear in the public record but are included in searches). Be as specific and precise as possible. Avoid acronyms and abbreviations unless these are in wide use.

Health coaching, pain, opioid medication, pain management, motivational interviewing, behavioral intervention, overdose

# Details of any existing review of the same topic by the same authors.

Give details of earlier versions of the systematic review if an update of an existing review is being registered, including full bibliographic reference if possible.

# * Current review status.

Review status should be updated when the review is completed and when it is published. For newregistrations the review must be Ongoing.

Please provide anticipated publication date Review_Ongoing

# Any additional information.

Provide any other information the review team feel is relevant to the registration of the review.

# Details of final report/publication(s).

This field should be left empty until details of the completed review are available. Give the link to the published review.
