# Supplementary material for: Low theoretical fidelity hinders the research on health coaching for opioid reduction: A systematic review of randomized controlled trials
Source: PLoS One. 2020 Oct 29;15(10):e0241434. doi: 10.1371/journal.pone.0241434 (PMC7595321; doi:10.1371/journal.pone.0241434)
Supplement: S2 Appendix — (DOCX) [file pone.0241434.s003.docx]

**S2 Appendix. Search terms used for electronic databases**

**Search strategy (June 5, 2019)**

Database: PubMed

| Set # |  | Results |
| --- | --- | --- |
| 1  Health coaching | "Health coaching"[tw] OR "health coaches"[tw] OR "health coach"[tw] OR "Motivational Interviewing"[Mesh] OR "Motivational interviewing"[tw] OR "motivational intervention"[tw] OR "motivational coaching"[tw] OR ((health[tiab] OR ehealth[tw] OR "Self Care"[Mesh] OR "behavior change"[tw]) AND ("Mentoring"[Mesh] OR mentoring[tw] OR coach[tw] OR coaching[tiab] OR coaches[tw] OR motivation[tw] OR motivational[tw])) | 32,531 |
| 2  opioids | "Analgesics, Opioid" [Pharmacological Action] OR "Analgesics, Opioid"[Mesh] OR "codeine"[MeSH Terms] OR "fentanyl"[MeSH Terms] OR "hydrocodone"[MeSH Terms] OR "acetaminophen, hydrocodone drug combination"[Supplementary Concept] OR "hydromorphone"[MeSH Terms] OR "meperidine"[MeSH Terms] OR "methadone"[MeSH Terms] OR "morphine"[MeSH Terms] OR "oxycodone"[MeSH Terms] OR "oxycodone-acetaminophen"[Supplementary Concept] OR "naloxone"[MeSH Terms] OR "Opiate Alkaloids"[Mesh] OR "Oxymorphone"[Mesh] OR “Narcotics” [Mesh] OR Opioid[tiab] OR opioids[tiab] OR opiate[tiab] OR opiates[tiab] OR "codeine"[tiab] OR "fentanyl"[tiab] OR atiq[tiab] OR "fentanyl"[tiab] OR "duragesic"[tiab] OR "fentora"[tiab] OR "hydrocodone"[tiab] OR "Hysingla ER"[tiab] OR "Zohydro ER"[tiab] OR "hydrocodone/acetaminophen"[tiab] OR loret[tiab] OR lortat[tiab] OR narco[tiab] OR "vicodin"[tiab] OR "hydromorphone"[tiab] OR "dilaudid"[tiab] OR exalto[tiab] OR "meperidine"[tiab] OR "demerol"[tiab] OR "methadone"[tiab] OR "morphine"[tiab] OR astromorph[tiab] OR avinza[tiab] OR kadian[tiab] OR "MS Contin"[tiab] OR "oxycodone"[tiab] OR "oxycontin"[tiab] OR oxecta[tiab] OR roxicodone[tiab] OR "oxycodone-acetaminophen"[tiab] OR "percocet"[tiab] OR endocert[tiab] OR toxicet[tiab] OR "naloxone"[tiab] OR Targiniq[tiab] OR "Oxymorphone"[tiab] OR “Narcotic”[tiab] OR narcotics[tiab])) OR "opioid-related disorders"[MeSH Terms] OR "analgesics, opioid"[Pharmacological Action] OR "analgesics, opioid"[MeSH Terms] OR "opioid"[tiab] OR "opioids"[tiab] OR Opioid[tiab] OR "narcotics"[Pharmacological Action] OR "narcotics"[MeSH Terms] OR "narcotics"[tiab] OR "narcotic"[tiab] OR "oxycodone"[MeSH Terms] OR "oxycodone"[tiab] OR "tramadol"[MeSH Terms] OR "tramadol"[tiab] OR "hydrocodone"[MeSH Terms] OR "hydrocodone"[tiab] OR "tapentadol"[Supplementary Concept] OR "tapentadol"[tiab] OR "Opioid-Related Disorders"[Mesh] OR "illicit drug use"[tiab] OR “illegal drug use”[tiab] OR "prescription drug misuse"[tiab] | 219,065 |
| 3 | 1 AND 2 | 564 |
| 4 | NOT (Editorial[pt] OR Letter[pt] OR Comment[pt]) | 559 |

15607841[uid] OR 7735025[uid]  OR 25096690[uid] OR 25159493[uid] OR 30249279[uid] OR 26083955[uid]

((((("Health coaching"[tw] OR "health coaches"[tw] OR "health coach"[tw] OR "Motivational Interviewing"[Mesh] OR "Motivational interviewing"[tw] OR "motivational intervention"[tw] OR "motivational coaching"[tw] OR ((health[tiab] OR ehealth[tw] OR "Self Care"[Mesh] OR "behavior change"[tw]) AND ("Mentoring"[Mesh] OR mentoring[tw] OR coach[tw] OR coaching[tiab] OR coaches[tw] OR motivation[tw] OR motivational[tw]))))) AND ((((("Analgesics, Opioid" [Pharmacological Action] OR "Analgesics, Opioid"[Mesh] OR "codeine"[MeSH Terms] OR "fentanyl"[MeSH Terms] OR "hydrocodone"[MeSH Terms] OR "acetaminophen, hydrocodone drug combination"[Supplementary Concept] OR "hydromorphone"[MeSH Terms] OR "meperidine"[MeSH Terms] OR "methadone"[MeSH Terms] OR "morphine"[MeSH Terms] OR "oxycodone"[MeSH Terms] OR "oxycodone-acetaminophen"[Supplementary Concept] OR "naloxone"[MeSH Terms] OR "Opiate Alkaloids"[Mesh] OR "Oxymorphone"[Mesh] OR “Narcotics” [Mesh] OR Opioid[tiab] OR opioids[tiab] OR opiate[tiab] OR opiates[tiab] OR "codeine"[tiab] OR "fentanyl"[tiab] OR atiq[tiab] OR "fentanyl"[tiab] OR "duragesic"[tiab] OR "fentora"[tiab] OR "hydrocodone"[tiab] OR "Hysingla ER"[tiab] OR "Zohydro ER"[tiab] OR "hydrocodone/acetaminophen"[tiab] OR loret[tiab] OR lortat[tiab] OR narco[tiab] OR "vicodin"[tiab] OR "hydromorphone"[tiab] OR "dilaudid"[tiab] OR exalto[tiab] OR "meperidine"[tiab] OR "demerol"[tiab] OR "methadone"[tiab] OR "morphine"[tiab] OR astromorph[tiab] OR avinza[tiab] OR kadian[tiab] OR "MS Contin"[tiab] OR "oxycodone"[tiab] OR "oxycontin"[tiab] OR oxecta[tiab] OR roxicodone[tiab] OR "oxycodone-acetaminophen"[tiab] OR "percocet"[tiab] OR endocert[tiab] OR toxicet[tiab] OR "naloxone"[tiab] OR Targiniq[tiab] OR "Oxymorphone"[tiab] OR “Narcotic”[tiab] OR narcotics[tiab])) OR ("opioid-related disorders"[MeSH Terms] OR "analgesics, opioid"[Pharmacological Action] OR "analgesics, opioid"[MeSH Terms] OR "opioid"[tiab] OR "opioids"[tiab] OR Opioid[tiab] OR "narcotics"[Pharmacological Action] OR "narcotics"[MeSH Terms] OR "narcotics"[tiab] OR "narcotic"[tiab] OR "oxycodone"[MeSH Terms] OR "oxycodone"[tiab] OR "tramadol"[MeSH Terms] OR "tramadol"[tiab] OR "hydrocodone"[MeSH Terms] OR "hydrocodone"[tiab] OR "tapentadol"[Supplementary Concept] OR "tapentadol"[tiab] OR "Opioid-Related Disorders"[Mesh] OR "illicit drug use"[tiab] OR “illegal drug use”[tiab] OR "prescription drug misuse"[tiab])))))) NOT ((Editorial[pt] OR Letter[pt] OR Comment[pt]))

Database: Embase

| Set # |  | Results |
| --- | --- | --- |
| 1  Health coaching | 'Health coaching':ti,ab OR 'health coaches':ti,ab OR 'health coach':ti,ab OR 'Motivational Interviewing'/de OR 'Motivational interviewing':ti,ab OR 'motivational intervention':ti,ab OR 'motivational coaching':ti,ab OR ((health:ti,ab OR ehealth:ti,ab OR 'Self Care'/de OR 'behavior change'/de OR 'behavior change':ti,ab) AND ('Mentoring'/de OR mentoring:ti,ab OR coach:ti,ab OR coaching:ti,ab OR coaches:ti,ab OR 'motivation'/de OR motivation:ti,ab OR motivational:ti,ab)) | 43,107 |
| 2  opioids | 'opiate'/de OR 'opiate addiction'/de OR 'codeine'/de OR 'fentanyl'/de OR 'hydrocodone'/de OR 'hydromorphone'/de OR 'pethidine'/de OR 'methadone'/de OR 'morphine derivative'/exp OR 'oxycodone'/de OR 'oxycodone-acetaminophen':ti,ab OR 'naloxone'/de OR 'opiate agonist'/de OR 'Opiate Alkaloids':ti,ab OR 'Oxymorphone'/de OR 'Narcotic agent'/exp OR 'narcotic analgesic agent'/exp OR 'oxycodone plus paracetamol'/de OR 'tramadol'/de OR 'tapentadol'/de OR Opioid:ti,ab OR opioids:ti,ab OR opiate:ti,ab OR opiates:ti,ab OR 'codeine':ti,ab OR 'fentanyl':ti,ab OR atiq:ti,ab OR 'duragesic':ti,ab OR 'fentora':ti,ab OR 'hydrocodone':ti,ab OR 'Hysingla ER':ti,ab OR 'Zohydro ER':ti,ab OR loret:ti,ab OR lortat:ti,ab OR narco:ti,ab OR 'vicodin':ti,ab OR 'hydromorphone':ti,ab OR 'dilaudid':ti,ab OR exalto:ti,ab OR 'meperidine':ti,ab OR 'demerol':ti,ab OR 'methadone':ti,ab OR 'morphine':ti,ab OR astromorph:ti,ab OR avinza:ti,ab OR kadian:ti,ab OR 'MS Contin':ti,ab OR 'oxycodone':ti,ab OR 'oxycontin':ti,ab OR oxecta:ti,ab OR roxicodone:ti,ab OR 'percocet':ti,ab OR endocert:ti,ab OR toxicet:ti,ab OR 'naloxone':ti,ab OR Targiniq:ti,ab OR 'Oxymorphone':ti,ab OR 'Narcotic':ti,ab OR narcotics:ti,ab OR 'tramadol':ti,ab OR 'hydrocodone':ti,ab OR 'tapentadol':ti,ab OR 'illicit drug use':ti,ab OR 'illegal drug use':ti,ab OR 'prescription drug misuse':ti,ab | 417,659 |
| 3 | 1 AND 2 | 929 |
| 4 | AND ('article'/it OR 'article in press'/it OR 'conference paper'/it OR 'review'/it) | 699 |

'Health coaching':ti,ab OR 'health coaches':ti,ab OR 'health coach':ti,ab OR 'Motivational Interviewing'/de OR 'Motivational interviewing':ti,ab OR 'motivational intervention':ti,ab OR 'motivational coaching':ti,ab OR ((health:ti,ab OR ehealth:ti,ab OR 'Self Care'/de OR 'behavior change'/de OR 'behavior change':ti,ab) AND ('Mentoring'/de OR mentoring:ti,ab OR coach:ti,ab OR coaching:ti,ab OR coaches:ti,ab OR 'motivation'/de OR motivation:ti,ab OR motivational:ti,ab)) AND ('opiate'/de OR 'opiate addiction'/de OR 'codeine'/de OR 'fentanyl'/de OR 'hydrocodone'/de OR 'hydromorphone'/de OR 'pethidine'/de OR 'methadone'/de OR 'morphine derivative'/exp OR 'oxycodone'/de OR 'oxycodone-acetaminophen':ti,ab OR 'naloxone'/de OR 'opiate agonist'/de OR 'Opiate Alkaloids':ti,ab OR 'Oxymorphone'/de OR 'Narcotic agent'/exp OR 'narcotic analgesic agent'/exp OR 'oxycodone plus paracetamol'/de OR 'tramadol'/de OR 'tapentadol'/de OR Opioid:ti,ab OR opioids:ti,ab OR opiate:ti,ab OR opiates:ti,ab OR 'codeine':ti,ab OR 'fentanyl':ti,ab OR atiq:ti,ab OR 'duragesic':ti,ab OR 'fentora':ti,ab OR 'hydrocodone':ti,ab OR 'Hysingla ER':ti,ab OR 'Zohydro ER':ti,ab OR loret:ti,ab OR lortat:ti,ab OR narco:ti,ab OR 'vicodin':ti,ab OR 'hydromorphone':ti,ab OR 'dilaudid':ti,ab OR exalto:ti,ab OR 'meperidine':ti,ab OR 'demerol':ti,ab OR 'methadone':ti,ab OR 'morphine':ti,ab OR astromorph:ti,ab OR avinza:ti,ab OR kadian:ti,ab OR 'MS Contin':ti,ab OR 'oxycodone':ti,ab OR 'oxycontin':ti,ab OR oxecta:ti,ab OR roxicodone:ti,ab OR 'percocet':ti,ab OR endocert:ti,ab OR toxicet:ti,ab OR 'naloxone':ti,ab OR Targiniq:ti,ab OR 'Oxymorphone':ti,ab OR 'Narcotic':ti,ab OR narcotics:ti,ab OR 'tramadol':ti,ab OR 'hydrocodone':ti,ab OR 'tapentadol':ti,ab OR 'illicit drug use':ti,ab OR 'illegal drug use':ti,ab OR 'prescription drug misuse':ti,ab) AND ('article'/it OR 'article in press'/it OR 'conference paper'/it OR 'review'/it)

Database: Scopus

| Set # |  | Results |
| --- | --- | --- |
| 1  Health coaching | TITLE-ABS-KEY({Health coaching} OR {health coaches} OR {health coach} OR {Motivational interviewing} OR {motivational intervention} OR {motivational coaching} OR ((health OR ehealth OR e-health OR {self care} OR {behavior change}) AND (mentoring OR coach OR coaching OR coaches OR motivation OR motivational))) | 78,086 |
| 2  opioids | TITLE-ABS-KEY(opioid OR opioids OR codeine OR fentanyl OR hydrocodone OR {acetaminophen hydrocodone} OR hydromorphone OR meperidine OR methadone OR morphine OR oxycodone OR {oxycodone-acetaminophen} OR {opiate alkaloids} OR Oxymorphone OR opiate OR opiates OR atiq OR duragesic OR fentora OR {Hysingla ER} OR {Zohydro ER} OR loret OR lortat OR narco OR vicodin OR dilaudid OR exalto OR meperidine OR demerol OR astromorph OR avinza OR kadian OR {MS Contin} OR oxycontin OR oxecta OR roxicodone OR percocet OR endocert OR toxicet OR naloxone OR Targiniq OR Narcotic OR narcotics OR {opioid-related disorder} OR {opioid-related disorders} OR tramadol OR tapentadol OR {illicit drug use} OR {illegal drug use} OR {prescription drug misuse}) | 362,064 |
| 3 | 1 AND 2 | 1,287 |
| 4 | AND (LIMIT-TO ( DOCTYPE , "ar" ) OR LIMIT-TO ( DOCTYPE , "re" ) OR LIMIT-TO ( DOCTYPE , "ip" ) ) | 1,179 |

(TITLE-ABS-KEY({Health coaching} OR {health coaches} OR {health coach} OR {Motivational interviewing} OR {motivational intervention} OR {motivational coaching} OR ((health OR ehealth OR e-health OR {self care} OR {behavior change}) AND (mentoring OR coach OR coaching OR coaches OR motivation OR motivational)))) AND (TITLE-ABS-KEY(opioid OR opioids OR codeine OR fentanyl OR hydrocodone OR {acetaminophen hydrocodone} OR hydromorphone OR meperidine OR methadone OR morphine OR oxycodone OR {oxycodone-acetaminophen} OR {opiate alkaloids} OR Oxymorphone OR opiate OR opiates OR atiq OR duragesic OR fentora OR {Hysingla ER} OR {Zohydro ER} OR loret OR lortat OR narco OR vicodin OR dilaudid OR exalto OR meperidine OR demerol OR astromorph OR avinza OR kadian OR {MS Contin} OR oxycontin OR oxecta OR roxicodone OR percocet OR endocert OR toxicet OR naloxone OR Targiniq OR Narcotic OR narcotics OR {opioid-related disorder} OR {opioid-related disorders} OR tramadol OR tapentadol OR {illicit drug use} OR {illegal drug use} OR {prescription drug misuse})) AND (LIMIT-TO ( DOCTYPE , "ar" ) OR LIMIT-TO ( DOCTYPE , "re" ) OR LIMIT-TO ( DOCTYPE , "ip" ) )

Database: PsycINFO

| Set # |  | Results |
| --- | --- | --- |
| 1  Health coaching | DE ("Motivational Interviewing" OR "Motivation training" OR (("health" OR "electronic health services" OR "self-care skills" OR "behavior change") AND ("mentor" OR "coaches" OR "coaching" OR "coaching psychology" OR "motivation"))) OR  TI (health coaching OR health coach OR health coaches OR motivational interviewing OR motivational intervention OR motivational coaching OR ((health OR ehealth OR e-health OR "self care" OR "behavior change") AND (mentoring OR coach OR coaches OR coaching OR motivation OR motivational))) OR AB (health coaching OR health coach OR health coaches OR motivational interviewing OR motivational intervention OR motivational coaching OR ((health OR ehealth OR e-health OR "self care" OR "behavior change") AND (mentoring OR coach OR coaches OR coaching OR motivation OR motivational))) | 21,277 |
| 2  opioids | DE ( "opiates" OR "opioid use disorder" OR "narcotic antagonists" Or "narcotic agonists" OR "codeine" OR "fentanyl" OR "meperidine" OR "methadone" OR "morphine" OR "oxycodone" OR "naloxone" OR “Narcotic drugs” OR "tramadol" ) OR TI ( opioid OR opioids OR codeine OR fentanyl OR hydrocodone OR "acetaminophen hydrocodone" OR hydromorphone OR meperidine OR methadone OR morphine OR oxycodone OR "oxycodone-acetaminophen" OR "opiate alkaloids" OR oxymorphone OR opiate OR opiates OR atiq OR duragesic OR fentora OR "Hysingla ER" OR "Zohydro ER" OR loret OR lortat OR narco OR vicodin OR dilaudid OR exalto OR meperidine OR demerol OR astromorph OR avinza OR kadian OR "MS Contin" OR oxycontin OR oxecta OR roxicodone OR percocet OR endocert OR toxicet OR naloxone OR Targiniq OR Narcotic OR narcotics OR "opioid-related disorder" OR “opioid-related disorders” OR tramadol OR tapentadol OR "illicit drug use" OR "illegal drug use" OR "prescription drug misuse" ) OR AB ( opioid OR opioids OR codeine OR fentanyl OR hydrocodone OR "acetaminophen hydrocodone" OR hydromorphone OR meperidine OR methadone OR morphine OR oxycodone OR "oxycodone-acetaminophen" OR "opiate alkaloids" OR oxymorphone OR opiate OR opiates OR atiq OR duragesic OR fentora OR "Hysingla ER" OR "Zohydro ER" OR loret OR lortat OR narco OR vicodin OR dilaudid OR exalto OR meperidine OR demerol OR astromorph OR avinza OR kadian OR "MS Contin" OR oxycontin OR oxecta OR roxicodone OR percocet OR endocert OR toxicet OR naloxone OR Targiniq OR Narcotic OR narcotics OR "opioid-related disorder" OR “opioid-related disorders” OR tramadol OR tapentadol OR "illicit drug use" OR "illegal drug use" OR "prescription drug misuse" ) | 45,295 |
| 3 | 1 AND 2 | 310 |
| 4 | Removed books | 277 |

( DE ( "opiates" OR "opioid use disorder" OR "narcotic antagonists" Or "narcotic agonists" OR "codeine" OR "fentanyl" OR "meperidine" OR "methadone" OR "morphine" OR "oxycodone" OR "naloxone" OR “Narcotic drugs” OR "tramadol" ) OR TI ( opioid OR opioids OR codeine OR fentanyl OR hydrocodone OR "acetaminophen hydrocodone" OR hydromorphone OR meperidine OR methadone OR morphine OR oxycodone OR "oxycodone-acetaminophen" OR "opiate alkaloids" OR oxymorphone OR opiate OR opiates OR atiq OR duragesic OR fentora OR "Hysingla ER" OR "Zohydro ER" OR loret OR lortat OR narco OR vicodin OR dilaudid OR exalto OR meperidine OR demerol OR astromorph OR avinza OR kadian OR "MS Contin" OR oxycontin OR oxecta OR roxicodone OR percocet OR endocert OR toxicet OR naloxone OR Targiniq OR Narcotic OR narcotics OR "opioid-related disorder" OR “opioid-related disorders” OR tramadol OR tapentadol OR "illicit drug use" OR "illegal drug use" OR "prescription drug misuse" ) OR AB ( opioid OR opioids OR codeine OR fentanyl OR hydrocodone OR "acetaminophen hydrocodone" OR hydromorphone OR meperidine OR methadone OR morphine OR oxycodone OR "oxycodone-acetaminophen" OR "opiate alkaloids" OR oxymorphone OR opiate OR opiates OR atiq OR duragesic OR fentora OR "Hysingla ER" OR "Zohydro ER" OR loret OR lortat OR narco OR vicodin OR dilaudid OR exalto OR meperidine OR demerol OR astromorph OR avinza OR kadian OR "MS Contin" OR oxycontin OR oxecta OR roxicodone OR percocet OR endocert OR toxicet OR naloxone OR Targiniq OR Narcotic OR narcotics OR "opioid-related disorder" OR “opioid-related disorders” OR tramadol OR tapentadol OR "illicit drug use" OR "illegal drug use" OR "prescription drug misuse" ) ) AND ( DE ("Motivational Interviewing" OR "Motivation training" OR (("health" OR "electronic health services" OR "self-care skills" OR "behavior change") AND ("mentor" OR "coaches" OR "coaching" OR "coaching psychology" OR "motivation"))) OR TI (health coaching OR health coach OR health coaches OR motivational interviewing OR motivational intervention OR motivational coaching OR ((health OR ehealth OR e-health OR "self care" OR "behavior change") AND (mentoring OR coach OR coaches OR coaching OR motivation OR motivational))) OR AB (health coaching OR health coach OR health coaches OR motivational interviewing OR motivational intervention OR motivational coaching OR ((health OR ehealth OR e-health OR "self care" OR "behavior change") AND (mentoring OR coach OR coaches OR coaching OR motivation OR motivational))) )

**Updated search strategy (December 10, 2019)**

Database: MEDLINE via PubMed

| Set # |  | Results |
| --- | --- | --- |
| 1  Health coaching | "Health coaching"[tw] OR "health coaches"[tw] OR "health coach"[tw] OR "Motivational Interviewing"[Mesh] OR "Motivational interviewing"[tw] OR "motivational intervention"[tw] OR "motivational coaching"[tw] OR ((health[tiab] OR ehealth[tw] OR "Self Care"[Mesh] OR "behavior change"[tw]) AND ("Mentoring"[Mesh] OR mentoring[tw] OR coach[tw] OR coaching[tiab] OR coaches[tw] OR motivation[tw] OR motivational[tw])) | 34,319 |
| 2  opioids | "Analgesics, Opioid" [Pharmacological Action] OR "Analgesics, Opioid"[Mesh] OR "codeine"[MeSH Terms] OR "fentanyl"[MeSH Terms] OR "hydrocodone"[MeSH Terms] OR "acetaminophen, hydrocodone drug combination"[Supplementary Concept] OR "hydromorphone"[MeSH Terms] OR "meperidine"[MeSH Terms] OR "methadone"[MeSH Terms] OR "morphine"[MeSH Terms] OR "oxycodone"[MeSH Terms] OR "oxycodone-acetaminophen"[Supplementary Concept] OR "naloxone"[MeSH Terms] OR "Opiate Alkaloids"[Mesh] OR "Oxymorphone"[Mesh] OR “Narcotics” [Mesh] OR Opioid[tiab] OR opioids[tiab] OR opiate[tiab] OR opiates[tiab] OR "codeine"[tiab] OR "fentanyl"[tiab] OR atiq[tiab] OR "fentanyl"[tiab] OR "duragesic"[tiab] OR "fentora"[tiab] OR "hydrocodone"[tiab] OR "Hysingla ER"[tiab] OR "Zohydro ER"[tiab] OR "hydrocodone/acetaminophen"[tiab] OR loret[tiab] OR lortat[tiab] OR narco[tiab] OR "vicodin"[tiab] OR "hydromorphone"[tiab] OR "dilaudid"[tiab] OR exalto[tiab] OR "meperidine"[tiab] OR "demerol"[tiab] OR "methadone"[tiab] OR "morphine"[tiab] OR astromorph[tiab] OR avinza[tiab] OR kadian[tiab] OR "MS Contin"[tiab] OR "oxycodone"[tiab] OR "oxycontin"[tiab] OR oxecta[tiab] OR roxicodone[tiab] OR "oxycodone-acetaminophen"[tiab] OR "percocet"[tiab] OR endocert[tiab] OR toxicet[tiab] OR "naloxone"[tiab] OR Targiniq[tiab] OR "Oxymorphone"[tiab] OR “Narcotic”[tiab] OR narcotics[tiab] OR "opioid-related disorders"[MeSH Terms] OR "analgesics, opioid"[Pharmacological Action] OR "analgesics, opioid"[MeSH Terms] OR "opioid"[tiab] OR "opioids"[tiab] OR Opioid[tiab] OR "narcotics"[Pharmacological Action] OR "narcotics"[MeSH Terms] OR "narcotics"[tiab] OR "narcotic"[tiab] OR "oxycodone"[MeSH Terms] OR "oxycodone"[tiab] OR "tramadol"[MeSH Terms] OR "tramadol"[tiab] OR "hydrocodone"[MeSH Terms] OR "hydrocodone"[tiab] OR "tapentadol"[Supplementary Concept] OR "tapentadol"[tiab] OR "Opioid-Related Disorders"[Mesh] OR "illicit drug use"[tiab] OR “illegal drug use”[tiab] OR "prescription drug misuse"[tiab] | 224,607 |
| 3 | 1 AND 2 | 614 |
| 4 | NOT (Editorial[pt] OR Letter[pt] OR Comment[pt]) | 605 |
| 5 | 4 AND  ("2019/06/01"[Date - MeSH] : "2021"[Date - MeSH]) | 68 |

Database: Embase via Elsevier

| Set # |  | Results |
| --- | --- | --- |
| 1  Health coaching | 'Health coaching':ti,ab OR 'health coaches':ti,ab OR 'health coach':ti,ab OR 'Motivational Interviewing'/de OR 'Motivational interviewing':ti,ab OR 'motivational intervention':ti,ab OR 'motivational coaching':ti,ab OR ((health:ti,ab OR ehealth:ti,ab OR 'Self Care'/de OR 'behavior change'/de OR 'behavior change':ti,ab) AND ('Mentoring'/de OR mentoring:ti,ab OR coach:ti,ab OR coaching:ti,ab OR coaches:ti,ab OR 'motivation'/de OR motivation:ti,ab OR motivational:ti,ab)) | 45,607 |
| 2  opioids | 'opiate'/de OR 'opiate addiction'/de OR 'codeine'/de OR 'fentanyl'/de OR 'hydrocodone'/de OR 'hydromorphone'/de OR 'pethidine'/de OR 'methadone'/de OR 'morphine derivative'/exp OR 'oxycodone'/de OR 'oxycodone-acetaminophen':ti,ab OR 'naloxone'/de OR 'opiate agonist'/de OR 'Opiate Alkaloids':ti,ab OR 'Oxymorphone'/de OR 'Narcotic agent'/exp OR 'narcotic analgesic agent'/exp OR 'oxycodone plus paracetamol'/de OR 'tramadol'/de OR 'tapentadol'/de OR Opioid:ti,ab OR opioids:ti,ab OR opiate:ti,ab OR opiates:ti,ab OR 'codeine':ti,ab OR 'fentanyl':ti,ab OR atiq:ti,ab OR 'duragesic':ti,ab OR 'fentora':ti,ab OR 'hydrocodone':ti,ab OR 'Hysingla ER':ti,ab OR 'Zohydro ER':ti,ab OR loret:ti,ab OR lortat:ti,ab OR narco:ti,ab OR 'vicodin':ti,ab OR 'hydromorphone':ti,ab OR 'dilaudid':ti,ab OR exalto:ti,ab OR 'meperidine':ti,ab OR 'demerol':ti,ab OR 'methadone':ti,ab OR 'morphine':ti,ab OR astromorph:ti,ab OR avinza:ti,ab OR kadian:ti,ab OR 'MS Contin':ti,ab OR 'oxycodone':ti,ab OR 'oxycontin':ti,ab OR oxecta:ti,ab OR roxicodone:ti,ab OR 'percocet':ti,ab OR endocert:ti,ab OR toxicet:ti,ab OR 'naloxone':ti,ab OR Targiniq:ti,ab OR 'Oxymorphone':ti,ab OR 'Narcotic':ti,ab OR narcotics:ti,ab OR 'tramadol':ti,ab OR 'hydrocodone':ti,ab OR 'tapentadol':ti,ab OR 'illicit drug use':ti,ab OR 'illegal drug use':ti,ab OR 'prescription drug misuse':ti,ab | 429,720 |
| 3 | 1 AND 2 | 994 |
| 4 | AND ('article'/it OR 'article in press'/it OR 'conference paper'/it OR 'review'/it) | 739 |
| 5 | 4 AND (2019:py OR 2020:py) | 61 |

Database: Scopus via Elsevier

| Set # |  | Results |
| --- | --- | --- |
| 1  Health coaching | TITLE-ABS-KEY({Health coaching} OR {health coaches} OR {health coach} OR {Motivational interviewing} OR {motivational intervention} OR {motivational coaching} OR ((health OR ehealth OR e-health OR {self care} OR {behavior change}) AND (mentoring OR coach OR coaching OR coaches OR motivation OR motivational))) | 82,109 |
| 2  opioids | TITLE-ABS-KEY(opioid OR opioids OR codeine OR fentanyl OR hydrocodone OR {acetaminophen hydrocodone} OR hydromorphone OR meperidine OR methadone OR morphine OR oxycodone OR {oxycodone-acetaminophen} OR {opiate alkaloids} OR Oxymorphone OR opiate OR opiates OR atiq OR duragesic OR fentora OR {Hysingla ER} OR {Zohydro ER} OR loret OR lortat OR narco OR vicodin OR dilaudid OR exalto OR meperidine OR demerol OR astromorph OR avinza OR kadian OR {MS Contin} OR oxycontin OR oxecta OR roxicodone OR percocet OR endocert OR toxicet OR naloxone OR Targiniq OR Narcotic OR narcotics OR {opioid-related disorder} OR {opioid-related disorders} OR tramadol OR tapentadol OR {illicit drug use} OR {illegal drug use} OR {prescription drug misuse}) | 370,742 |
| 3 | 1 AND 2 | 1,360 |
| 4 | AND (LIMIT-TO ( DOCTYPE , "ar" ) OR LIMIT-TO ( DOCTYPE , "re" ) OR LIMIT-TO ( DOCTYPE , "cp" ) ) | 1,266 |
| 5 | AND  ( LIMIT-TO ( PUBYEAR ,  2020 )  OR  LIMIT-TO ( PUBYEAR ,  2019 ) | 79 |

Database: PsycINFO via EBSCO

| Set # |  | Results |
| --- | --- | --- |
| 1  Health coaching | DE ("Motivational Interviewing" OR "Motivation training" OR (("health" OR "electronic health services" OR "self-care skills" OR "behavior change") AND ("mentor" OR "coaches" OR "coaching" OR "coaching psychology" OR "motivation"))) OR  TI (health coaching OR health coach OR health coaches OR motivational interviewing OR motivational intervention OR motivational coaching OR ((health OR ehealth OR e-health OR "self care" OR "behavior change") AND (mentoring OR coach OR coaches OR coaching OR motivation OR motivational))) OR AB (health coaching OR health coach OR health coaches OR motivational interviewing OR motivational intervention OR motivational coaching OR ((health OR ehealth OR e-health OR "self care" OR "behavior change") AND (mentoring OR coach OR coaches OR coaching OR motivation OR motivational))) | 22,044 |
| 2  opioids | DE ( "opiates" OR "opioid use disorder" OR "narcotic antagonists" Or "narcotic agonists" OR "codeine" OR "fentanyl" OR "meperidine" OR "methadone" OR "morphine" OR "oxycodone" OR "naloxone" OR “Narcotic drugs” OR "tramadol" ) OR TI ( opioid OR opioids OR codeine OR fentanyl OR hydrocodone OR "acetaminophen hydrocodone" OR hydromorphone OR meperidine OR methadone OR morphine OR oxycodone OR "oxycodone-acetaminophen" OR "opiate alkaloids" OR oxymorphone OR opiate OR opiates OR atiq OR duragesic OR fentora OR "Hysingla ER" OR "Zohydro ER" OR loret OR lortat OR narco OR vicodin OR dilaudid OR exalto OR meperidine OR demerol OR astromorph OR avinza OR kadian OR "MS Contin" OR oxycontin OR oxecta OR roxicodone OR percocet OR endocert OR toxicet OR naloxone OR Targiniq OR Narcotic OR narcotics OR "opioid-related disorder" OR “opioid-related disorders” OR tramadol OR tapentadol OR "illicit drug use" OR "illegal drug use" OR "prescription drug misuse" ) OR AB ( opioid OR opioids OR codeine OR fentanyl OR hydrocodone OR "acetaminophen hydrocodone" OR hydromorphone OR meperidine OR methadone OR morphine OR oxycodone OR "oxycodone-acetaminophen" OR "opiate alkaloids" OR oxymorphone OR opiate OR opiates OR atiq OR duragesic OR fentora OR "Hysingla ER" OR "Zohydro ER" OR loret OR lortat OR narco OR vicodin OR dilaudid OR exalto OR meperidine OR demerol OR astromorph OR avinza OR kadian OR "MS Contin" OR oxycontin OR oxecta OR roxicodone OR percocet OR endocert OR toxicet OR naloxone OR Targiniq OR Narcotic OR narcotics OR "opioid-related disorder" OR “opioid-related disorders” OR tramadol OR tapentadol OR "illicit drug use" OR "illegal drug use" OR "prescription drug misuse" ) | 46,453 |
| 3 | 1 AND 2 | 333 |
| 4 | Removed books | 299 |
| 5 | Publication Year: 2019-2021 | 21 |
