# Supplementary material for: Low theoretical fidelity hinders the research on health coaching for opioid reduction: A systematic review of randomized controlled trials
Source: PLoS One. 2020 Oct 29;15(10):e0241434. doi: 10.1371/journal.pone.0241434 (PMC7595321; doi:10.1371/journal.pone.0241434)
Supplement: S3 Appendix — (DOCX) [file pone.0241434.s004.docx]

**S3 Appendix. The GRADE approach to evidence synthesis [18]**

We categorized the certainty of evidence as follows:

- High Quality (⊕⊕⊕⊕): further research is very unlikely to change the confidence in the estimate of effect.
- Moderate Quality (⊕⊕⊕⊝): further research is likely to have an important impact in the confidence in the estimate of effect and may change the estimate.
- Low Quality (⊕⊕⊝⊝): further research is very likely to have an important impact on our confidence in the estimate of effect and is likely to change the estimate.
- Very Low Quality (⊕⊝⊝⊝): any estimate of effect is very uncertain and has very little confidence.

We graded the evidence available to answer each sub-question on the domains following criteria:

**1. Risk of bias**

Confidence in the estimate of the effect decreases if studies have major limitations in design and conduct. We assessed five types of bias:

- Risk of bias arising from the randomization process
- Risk of bias due to deviations from the intended interventions (effect of assignment to intervention)
- Missing outcome data
- Risk of bias in measurement of the outcome
- Risk of bias in selection of reported result

The certainty of evidence was downgraded one level for an estimate of effect that relied on studies with a high risk of bias in one of these domains. The certainty of evidence was downgraded by two levels for an estimate of effect that relied heavily on studies with a high risk of bias in two or more of these domains. The certainty of evidence was downgraded by one level for an estimate of effect that relied on studies with some concern in one of these domains. The certainty of evidence was downgraded by two levels for an estimate of effect that relied heavily on studies with some concern of bias in three or more of these domains.

**2. Indirectness**

Indirectness refers to a mismatch between the population, intervention, comparator, or outcomes for the studies included in the review and the population (adult participants older than or equal to 18 years old, either sex with pain, with or without a substance use disorder diagnosis), intervention (health coaching), comparator (education, usual care, and other psychological interventions), or outcomes (opioid use, pain intensity, physical function, and quality of life) for the research question being posed by the systematic review. We downgraded by one level if the intervention did not include all four constructs of health coaching. The certainty of evidence was downgraded by one level when there was indirectness for one element of the research question and by two levels when there was indirectness for two or more elements of the research question.

**3. Imprecision**

Imprecision refers to uncertainty in the results due to few participants or to wide confidence intervals. We used the following guidance in judging imprecision:

**For continuous outcomes**An outcome was downgraded one level for imprecision if:

- the total number of participants was fewer than 400; or
- the 95% confidence interval around the estimate of effect covered both no effect and a minimally important difference for that outcome, or if a minimally important difference was not prespecified, no effect and a standardized mean difference (SMD) of ± 0.5.

An outcome was downgraded two levels for imprecision if both points above were true.

**For dichotomous outcomes**

An outcome was downgraded one level for imprecision if:

- the total number of events was less than 300; or
- the 95% confidence interval around the estimate of effect included both no effect and either appreciable benefit or appreciable harm. The threshold for ’appreciable benefit’ or ’appreciable harm’ was a relative risk reduction (RRR) or relative risk increase (RRI) greater than 25%.

An outcome was downgraded two levels for imprecision if both points above were true.
