# Supplementary material for: Low theoretical fidelity hinders the research on health coaching for opioid reduction: A systematic review of randomized controlled trials
Source: PLoS One. 2020 Oct 29;15(10):e0241434. doi: 10.1371/journal.pone.0241434 (PMC7595321; doi:10.1371/journal.pone.0241434)
Supplement: S4 Appendix — (DOCX) [file pone.0241434.s005.docx]

**S4 Appendix. List of excluded studies/reasons for exclusion**

1. Aharonovich, E., Sarvet, A., Stohl, M., DesJarlais, D., Tross, S., Hurst, T., ... & Hasin, D. (2017). Reducing non-injection drug use in HIV primary care: a randomized trial of brief motivational interviewing, with and without HealthCall, a technology-based enhancement. *Journal of substance abuse treatment*, *74*, 71-79.

Reason for exclusion: Wrong outcome, opioid use is not an outcome.

1. Aldridge, A., Dowd, W., & Bray, J. (2017). The relative impact of brief treatment versus brief intervention in primary health‐care screening programs for substance use disorders. *Addiction*, *112*, 54-64.

Reason for exclusion: Other study design.

1. Bagley, S. M., Peterson, J., Cheng, D. M., Jose, C., Quinn, E., O’Connor, P. G., & Walley, A. Y. (2015). Overdose education and naloxone rescue kits for family members of individuals who use opioids: characteristics, motivations, and naloxone use. *Substance abuse*, *36*(2), 149-154.

Reason for exclusion: Wrong patient population.

1. Baker, A., Lewin, T., Reichler, H., Clancy, R., Carr, V., Garrett, R., ... & Terry, M. (2002). Evaluation of a motivational interview for substance use within psychiatric in‐patient services. *Addiction*, *97*(10), 1329-1337.

Reason for exclusion: Other study design.

1. Becka, J. (2004). Efficacy of Cognitive Behavioral Methods in Decreasing Drug Use by Methadone Clinic Patients Compared with Standard Conditions. *CESKA A SLOVENSKA PSYCHIATRIE*, *100*, 4-7.

Reason for exclusion: Wrong intervention.

1. Bellack, A. S., Bennett, M. E., Gearon, J. S., Brown, C. H., & Yang, Y. (2006). A randomized clinical trial of a new behavioral treatment for drug abuse in people with severe and persistent mental illness. *Archives of general psychiatry*, *63*(4), 426-432.

Reason for exclusion: Wrong intervention.

1. Bhimani, R. H., Cross, L. J., Taylor, B. C., Meis, L. A., Fu, S. S., Allen, K. D., ... & Burgess, D. J. (2017). Taking ACTION to reduce pain: ACTION study rationale, design and protocol of a randomized trial of a proactive telephone-based coaching intervention for chronic musculoskeletal pain among African Americans. *BMC musculoskeletal disorders*, *18*(1), 15.

Reason for exclusion: Wrong intervention.

1. Bohnert, A. S., Bonar, E. E., Cunningham, R., Greenwald, M. K., Thomas, L., Chermack, S., ... & Walton, M. (2016). A pilot randomized clinical trial of an intervention to reduce overdose risk behaviors among emergency department patients at risk for prescription opioid overdose. *Drug and alcohol dependence,*163, 40-47.

Reason for exclusion: Wrong outcome.

1. Bundalo-Vrbanac, D., Buljan, D., Peitl, V., & Gelo, J. (2012). Integrating psychotherapy and pharmacotherapy in treatment of substance dependence. *Alcoholism and Psychiatry Research*, *48*(2), 107.

Reason for exclusion: Other study design.

1. Chang, Y. P., Compton, P., Almeter, P., & Fox, C. H. (2015). The Effect of Motivational Interviewing on Prescription Opioid Adherence Among Older Adults With Chronic Pain. *Perspectives in psychiatric care*, *51*(3), 211-219.

Reason for exclusion: Other study design.

1. Christensen, D. R., Landes, R. D., Jackson, L., Marsch, L. A., Mancino, M. J., Chopra, M. P., & Bickel, W. K. (2014). Adding an Internet-delivered treatment to an efficacious treatment package for opioid dependence. *Journal of consulting and clinical psychology*, *82*(6), 964.

Reason for exclusion: Wrong intervention.

1. Cochran, G.; Chen, Q.; Field, C.; Seybert, A. L.; Hruschak, V.; Jaber, A.; Gordon, A. J.; Tarter, R. (2019). A community pharmacy-led intervention for opioid medication misuse: A small-scale randomized clinical trial. *Drug and alcohol dependence*, 205():107570

Reason for exclusion: Other study design.

1. Crawley, A., Murphy, L., Regier, L., & McKee, N. (2018). Tapering opioids using motivational interviewing. *Canadian Family Physician*, *64*(8), 584-587.

Reason for exclusion: Other study design.

1. Cummins, D. M.; Tobian, R. (2018). Motivational Enhancement Therapy for Veterans with Chronic Pain and Substance Use. *Health & Social Work*, 43(4):269-273

Reason for exclusion: Other study design.

1. Darker, C. D., Sweeney, B., Keenan, E., Whiston, L., Anderson, R., & Barry, J. (2016). Screening and brief interventions for illicit drug use and alcohol use in methadone maintained opiate-dependent patients: results of a pilot cluster randomized controlled trial feasibility study. *Substance use & misuse*, *51*(9), 1104-1115.

Reason for exclusion: Wrong outcome.

1. Day, E., Copello, A., Seddon, J. L., Christie, M., Bamber, D., Powell, C., ... & Freemantle, N. (2013). Pilot study of a social network intervention for heroin users in opiate substitution treatment: study protocol for a randomized controlled trial. *Trials*, *14*(1), 264.

Reason for exclusion: Wrong intervention.

1. Forray, A., Martino, S., Gilstad-Hayden, K., Kershaw, T., Ondersma, S., Olmstead, T., & Yonkers, K. A. (2019). Assessment of an electronic and clinician-delivered brief intervention on cigarette, alcohol and illicit drug use among women in a reproductive healthcare clinic. *Addictive behaviors*, *96*, 156-163.

Reason for exclusion: Other study design.

1. Humeniuk, R., Ali, R., Babor, T., Souza‐Formigoni, M. L. O., de Lacerda, R. B., Ling, W., ... & Simon, S. (2012). A randomized controlled trial of a brief intervention for illicit drugs linked to the Alcohol, Smoking and Substance Involvement Screening Test (ASSIST) in clients recruited from primary health‐care settings in four countries. *Addiction*, *107*(5), 957-966.

Reason for exclusion: Wrong patient population, includes those under 18.

1. Kavanagh, D. J., Young, R., White, A., Saunders, J. B., Wallis, J., Shockley, N., ... & Clair, A. (2004). A brief motivational intervention for substance misuse in recent‐onset psychosis. *Drug and Alcohol Review*, *23*(2), 151-155.

Reason for exclusion: Wrong patient population, includes those under 18.

1. Kazemi, D. M., Levine, M. J., Dmochowski, J., Shou, Q., & Angbing, I. (2013). Brief motivational intervention for high-risk drinking and illicit drug use in mandated and voluntary freshmen. *Journal of Substance Use*, *18*(5), 392-404.

Reason for exclusion: Other study design.

1. Kim, T. W., Bernstein, J., Cheng, D. M., Lloyd‐Travaglini, C., Samet, J. H., Palfai, T. P., & Saitz, R. (2017). Receipt of addiction treatment as a consequence of a brief intervention for drug use in primary care: a randomized trial. *Addiction*, *112*(5), 818-827.

Reason for exclusion: Other study design.

1. Longshore, D., & Grills, C. (2000). Motivating illegal drug use recovery: Evidence for a culturally congruent intervention. *Journal of Black Psychology*, *26*(3), 288-301.

Reason for exclusion: Wrong intervention.

1. Marsden, J., Stillwell, G., Hellier, J., Brown, A. M., Byford, S., Kelleher, M., ... & Mitcheson, L. (2017). Effectiveness of adjunctive, personalised psychosocial intervention for non-response to opioid agonist treatment: Study protocol for a pragmatic randomised controlled trial. *Contemporary clinical trials*, *53*, 36-43.

Reason for exclusion: Wrong intervention.

1. Martino, S., Ondersma, S. J., Forray, A., Olmstead, T. A., Gilstad-Hayden, K., Howell, H. B., ... & Yonkers, K. A. (2018). A randomized controlled trial of screening and brief interventions for substance misuse in reproductive health. *American journal of obstetrics and gynecology*, *218*(3), 322-e1.

Reason for exclusion: Wrong outcome, opioids use not an outcome.

1. Martino, S.; Zimbrean, P.; Forray, A.; Kaufman, J. S.; Desan, P. H.; Olmstead, T. A.; Gilstad-Hayden, K.; Gueorguieva, R.; Yonkers, K. A. (2019). Implementing Motivational Interviewing for Substance Misuse on Medical Inpatient Units: a Randomized Controlled Trial. *Journal of General Internal Medicine*, 34(11):2520-2529.

Reason for exclusion: Wrong outcome, opioid use not an outcome.

1. Ondersma, S. J., Svikis, D. S., Thacker, L. R., Beatty, J. R., & Lockhart, N. (2014). Computer-delivered screening and brief intervention (e-SBI) for postpartum drug use: a randomized trial. *Journal of substance abuse treatment*, *46*(1), 52-59.

Reason for exclusion: Wrong intervention.

1. Rustøen, T., Valeberg, B. T., Kolstad, E., Wist, E., Paul, S., & Miaskowski, C. (2014). A randomized clinical trial of the efficacy of a self-care intervention to improve cancer pain management. *Cancer nursing*, *37*(1), 34-43.

Reason for exclusion: Wrong intervention.

1. Satre, D. D., Delucchi, K., Lichtmacher, J., Sterling, S. A., & Weisner, C. (2013). Motivational interviewing to reduce hazardous drinking and drug use among depression patients. *Journal of substance abuse treatment*, *44*(3), 323-329.

Reason for exclusion: Wrong outcome, opioid use not an outcome.

1. Seal, K. H., Borsari, B., Tighe, J., Cohen, B. E., Delucchi, K., Morasco, B. J., ... & Manuel, J. K. (2019). Optimizing pain treatment interventions (OPTI): A pilot randomized controlled trial of collaborative care to improve chronic pain management and opioid safety—Rationale, methods, and lessons learned. *Contemporary clinical trials*, *77*, 76-85.

Reason for exclusion: Wrong outcome, opioid use not reported.

1. Secades-Villa, R., Ramón Fernánde-Hermida, J., & Arnáez-Montaraz, C. (2004). Motivational interviewing and treatment retention among drug user patients: a pilot study. *Substance Use & Misuse*, *39*(9), 1369-1378.

Reason for exclusion: Wrong outcome.

1. Shetty, V., Murphy, D. A., Zigler, C., Yamashita, D. D. R., & Belin, T. R. (2011). Randomized controlled trial of personalized motivational interventions in substance using patients with facial injuries. *Journal of Oral and Maxillofacial Surgery*, *69*(9), 2396-2411.

Reason for exclusion: Wrong outcome, opioid use not an outcome.

1. Woodruff, S. I., Clapp, J. D., Eisenberg, K., McCabe, C., Hohman, M., Shillington, A. M., ... & Gareri, J. (2014). Randomized clinical trial of the effects of screening and brief intervention for illicit drug use: the life shift/shift gears study. *Addiction science & clinical practice*, *9*(1), 8.

Reason for exclusion: Wrong intervention.

1. Zahradnik, A., Otto, C., Crackau, B., Löhrmann, I., Bischof, G., John, U., & Rumpf, H. J. (2009). Randomized controlled trial of a brief intervention for problematic prescription drug use in non‐treatment‐seeking patients. *Addiction*, *104*(1), 109-117.

Reason for exclusion: Wrong outcome, opioid use not an outcome.
