# Supplementary material for: Low theoretical fidelity hinders the research on health coaching for opioid reduction: A systematic review of randomized controlled trials
Source: PLoS One. 2020 Oct 29;15(10):e0241434. doi: 10.1371/journal.pone.0241434 (PMC7595321; doi:10.1371/journal.pone.0241434)
Supplement: S5 Appendix — (DOCX) [file pone.0241434.s006.docx]

**S5 Appendix. Description of included studies**

| **Author (year)** | **Country/Setting** | **Number of Participants (N)** | **Health Condition/**  **Population** | **Age (years) (mean and SD)** | **Sex (n and %)** | **Intervention** | **Comparison** | **Number of Sessions/**  **Duration of Treatment** | **Primary Outcomes** | **Secondary Outcomes** |
| --- | --- | --- | --- | --- | --- | --- | --- | --- | --- | --- |
| Bagoien (2013) | Norway/Psychiatric Emergency Unit | 135 | Emergency psychiatric in-patient patients, over 18, under the influence of a drug on admittance not prescribed by a physician. | Intervention: 36.9 (14), Control: 36.1 (13.4) | Male: Intervention - 38 (56.7%) Control - 40 (58.8%) Female: Intervention - 29 (43.3%), Control - 28 (41.2%) | Two sessions of manual guided motivational interviewing delivered individually to the patients by a trained therapist. | Treatment as usual included detoxification, pharmacotherapy, and general psychotherapy. General information about the harmful effects of substances and suggestions regarding treatment for substance use, including possible referral to specialty substance use treatment institutions, would be given. Planning of discharge with a referral to out-patient facility and primary community health care would be included. | Two sessions for 45 minutes each | Self-reported days per month of substance use **(**alcohol, amphetamines, benzodiazepines, cannabinoids, ecstasy, opioids**)** prior to and after an intervention. | Not reported |
| Bernstein (2005) | United States of America/ Outpatient Clinic | 778 | Over 18, use of cocaine or heroine in the last 30 days. | Intervention: 37.8 (8.3), Control: 381. (8.2) | Male: Intervention - 409 (69.4%), Control - 420 (71.8%) Female: Intervention - 181(30.6%), Control - 165(28.2%) | Semi-scripted brief motivational interview delivered by a peer (a substance abuse outreach worker in recovery). It was tailored to individual behavior, risks, culture, and language and if the patient desired a referral was given. One booster call was given later, with information given about referral if it was inquired about. | The control group received only the handout from the interventionist stating that “based on your screening responses, you would benefit from help with your drug use.” This form included a list of treatment options including detox, AA/NA, acupuncture, residential treatment facilities, and harm reduction information about safe sex and needle exchange, but there was no discussion about this information. | One 20 minute session with 5-10 min "booster" call ten days later | Cocaine and/or heroin use as measured by radioimmune assay of hair (RIA). | Not reported |
| Carroll (2006) | United States of America/ Outpatient Clinic | 423 | Those seeking outpatient treatment for any substance use problem and had used alcohol or any illicit drug at least once in the prior 28 days. Were 18 years of age or older, and were willing to participate in the protocol (e.g., to be randomized to treatment, be contacted for follow-up assessment, and to have their session audiotaped). | Total: 32.8 (9.9) | Male: Total: 240 (56.8%) Female: Total: 183 (43.2%) | Those in the motivational interviewing condition had a 2 hour assessment/evaluation where the therapist did the same intake session yet incorporated MI strategies (empathy, removing barriers, providing feedback, etc.) and used an MI interviewing style (asking open-ended questions, reflective listening, eliciting self-motivation, etc.) | Participants assigned to standard intake/eval were given a 2 hour assessment/eval where standard information was gathered. This typically included the patient's history, current level of substance use, treatment history and psychosocial function. A clinic orientation was completed and then participants were then referred to standard group treatment. | One session for a one hour motivational interview intake | Use of illicit drugs or substance abuse prior to and following motivational interviewing sessions | Psychosocial difficulties, quality of life in terms of how the drug affected them: medically, legally, employment, family wise, and psychologically. |
| Coffin (2017) | United States of America/ Naloxone distribution program centers - Drug Overdose Prevention and Education Program | 63 | 18–65 years of age; were opioid dependent by Structured Clinical Interview for the DSM IV (SCID); had an opioid overdose in the preceding 5 years and had previously received take-home naloxone, were able/willing to provide informed consent, communicate in English, and adhere to the visit schedule. | Overall: 43.3 (11.70) | Male: Intervention - 32 (74.4%), Control - 10 (50%) Female: Intervention - 11 (25.6%), Control - 10 (50%) | Counselors delivered a 45-minute intervention (REBOOT) based on the information-motivation-behavior skills model of behavior change. Counselors reviewed opioid overdose risk factors and response, then discussed personal and witnessed overdose events in detail with the participant to help them identify risk behaviors associated with the overdose. Counselors then asked about the participant’s interest in substance use disorder treatment and assisted in developing a plan to reduce the risk of future overdoses. HIV and HCV risk behavior and risk reduction strategies were also addressed. | Treatment as usual was a packet of information provided at baseline and follow up visits about harm reduction sites and substance use disorder treatment. There was also an offer to assist with referrals to any services the participant requested. | 4 sessions in 16 months | Opioid use prior to and after a behavioral intervention, including nonfatal opioid overdose within 5 years | Not reported |
| Jaffray (2014) | Scotland/Methadone Treatment Center | 542 | Participating pharmacists approached all daily supervised methadone patients, initiated in the last 24 months who were greater than 18 years of age. Patients were recruited both retrospectively (from the last 12 patients joining the pharmacy) and prospectively (patients starting methadone of the next 6 months) | 32.4 (7.2) | Male: 345 (63.7%) Female: 197 (36.3%) | Intervention pharmacists were trained in MI techniques during 4 sessions provided by Scottis Training on Drugs and Alcohol (STRATA) certified trainers. This training provided a framework for increased communication skills throughout the study by teaching the value of open ended questions and educating the pharmacist on alternative pain management methods that can be voiced to the patient. The intervention of MI was intended to be spread over a number of visits, building on discussions from previous interactions Discussions between pharmacist/patient were to be focused on reducing drug use, specifically illicit heroin. All groups whether intervention or control received four newsletters which provided study progress information, and the intervention group received MI technique reminders. | Those who were allocated to the control group had normal interactions with their pharmacists. They received four newsletters regarding the progress of the study, however, there were NO MI techniques noted. These individuals were assessed in face-to-face interviewing and were asked to fill out the MAP at baseline and 6 months as well as a satisfaction survey after completion of the study. | Baseline & 6 month follow up. However, the intervention was intended to be "spread through a number of visits" depending on how often one visited the pharmacy | illicit heroin use prior to and following motivational interviewing techniques | Psychological/physical health |
| Jamison (2010) | United States of America/ Outpatient Clinic | 62 | Noncancer related back pain who showed risk potential for or demonstration of opioid misuse. Patients had to have chronic back pain for greater than 6 months with a rating averaged 4 or greater on a pain intensity scale from 0-10 with medication, speak and understand English, had been prescribed opioid therapy for greater than 6 months, had risk for or history of prescription opioid misuse based on past records of abnormal urine screens and physician reports. RISK was determined by a tool called "Screener for Opioid Abuse for Pain Patients" (SOAPP-R) with a score greater than 18. | 47.7 (7.14) | Male: 56.5% Female: 43.5% | Those in the intervention group were maintained on their current opioid regime and were seen on a monthly basis at a pain treatment center where they completed electronic diaries and participated in a structured cognitive behavioral training program for prevention of substance misuse. As part of the interventions, they (1) participated in one or more group sessions in which risk factors regarding opioid use were discussed, (2) received monthly individual monthly motivational counseling sessions to review compliance issues, (3) were given substance misuse and education worksheets, (4), completed a monthly opioid compliance checklist developed specifically for this study, (5) had monthly urine screens. These documents and the urine analysis were performed monthly. In order for this group to get their next assigned prescription, all of these tasks had to have been completed. | Those in the high-risk control group were maintained on their current opioid regimen and were seen on a monthly basis at a pain treatment center at a university-based medical center. They completed electronic diaries and had monthly contact with their physician. They represented the usual treatment control condition. | Monthly for 6 months: Electronic diaries, urine screens, opioid compliance checklist, group education sessions, and individual motivational compliance counseling. | Opioid use prior to and after cognitive behavioral substance misuse counseling | Pain, activity, mood, and quality of life |
| Merchant (2015) | United States of America/ Emergency Department | 1,030 | Random sample of individuals in the ER. The inclusion/exclusion criteria attempted to mirror the general adult ED population who would be included in an SBIRT program. Those 18-64 year old English and/or Spanish speaking adults who were not critically injured or ill, not on house arrest or in prison, not presenting with psychological illnesses, not requesting treatment for substance abuse or misuse, not intoxicated, and having no physical or mental impairments from preventing them from being in the study were potentially eligible. Patients had to have an ASSIST (Alcohol, Smoking, and Substance Involvement Screening Test) score of at least 4. | 30 (not reported) | Male: 556 (54%) Female: 474 (46% | Following enrollment, participants were randomly assigned 1:1 into the two study arms (treatment vs control) using block randomization with a block size of 6. Those in the intervention group were assigned to receive BI, which was defined as "a technique to motivate patients to reduce their drug misuse and seek appropriate treatment". The BI sessions were approximately 20-30 minutes in length. Research assistants proctored the BI's and used motivational interviewing techniques (ex: decisional balance, and discussing goals and values) to facilitate a discussion about behavior changes. Each participant in the intervention group was contacted every 2-4 weeks after enrollment to have progress discussed and to review all the goals that were outlined in the initial BI session. Follow ups were done at 3 months using self-reported questionnaires online. (for clarity each patient only got ONE BI session) | Simple discharge from the ER with no BI techniques performed | One BI session 20 to 30 minutes long. Follow ups every 2-4 weeks to check in and re-assess goals discussed within the BI session (contacted telephone for booster session) | Alcohol, smoking, substance use (illicit opioids, marijuana, cocaine or "crack", methamphetamines, inhalants, hallucinogens, GHB, amphetamines, benzodiazepines, barbiturates, methadone, prescription opioids) prior to and after a brief intervention | Not reported |
| Nyamathi (2011) | United States of America/ Methadone Treatment Center | 256 | Received methadone for at least three months, were 18-55 years of age, reported moderate to heavy alcohol use. | 51.2 (8.4) | Male: 151 (59%) Female: 105 (41%) | Individual MI - not given detail, just say been "effective" | Group MI and nurse-Led hepatitis health promotion (HHP) | 3 sessions each | Reduction of drug (illicit opioids) use following a motivational interviewing session | Perceived health status, emotional well-being (quality of life) |
| Saitz (2014) | United States of America/ Primary Care Internal Medicine | 528 | Patients who screened positive on ASSIST by the health educators were referred immediately to research assistants. Research assistants completed further screening for study eligibility. | 41.3 (12) | Male: 369 (70.7%) Female: 159 (29.3%) | MOTIV - based on MI and elicited possible links between drug use and health concerns; made aware of negative outcomes and valued goals which enhances self efficacy about behavior change. BNI structured interview conducted by health educators that uses some features of MI and includes feedback, review of pros and cons of use and development of plan for change and provides options for change. | No intervention | MOTIV - 30 to 45 minutes with 20 to 30 minute booster session BNI - 10 to 15 minute | Illicit opioid or unhealthy drug use prior to and following a brief negotiated interview and an adaptation of motivational interviewing | Not reported |
| Saunders (1995) | Australia/Methadone Treatment Center | 122 | Opioid Use Disorder | 28 | Male: 79 (64.8%) Female: 43 (35.2%) | Motivational interviewing where the patients were invited to talk about positive aspects of their opiate use and then talk about negative consequences. | Education procedure where they patients were given a booklet containing information on opioid drugs. | One session for one hour | Illicit drug use (opioid use) following a brief motivational intervention. | Not reported |
| Zhong (2015) | China/Released from compulsatory rehab center and joined community rehabilitation | 180 | All patients had just been released from a compulsory treatment center, and joined the 3-year community rehabilitation program. | 38.7 (8.86) | Male: 141 (78.3%) Female: 39 (21.7%) | Participants randomly assigned to CPI group or usual community care group. The CPI adopted CBT theory, MI techniques, case management, and urine testing. MI explored participants' current situation and expectations. | Usual community care (UCC) group received monthly visits by social worker, urine tests, and simple advice regarding life events. | 60 minute session once a week for 1 year. CPI group received average of 19.9 individual sessions and took part in average of 3 group sessions Group sessions: once every 2 months, 90-100 minutes each. UCC group received monthly visits. | Opioid use following a year-long comprehensive psychosocial intervention | Quality of life and mental health |
